# New Alien Plant Taxa for Italy and Europe: An Update

**DOI:** 10.3390/plants13050620

**Published:** 2024-02-24

**Authors:** Carmelo Maria Musarella, Valentina Lucia Astrid Laface, Claudia Angiolini, Gianluigi Bacchetta, Enrico Bajona, Enrico Banfi, Giulio Barone, Nello Biscotti, Daniele Bonsanto, Giacomo Calvia, Salvatore Cambria, Alberto Capuano, Giuseppe Caruso, Alessandro Crisafulli, Emanuele Del Guacchio, Emilio Di Gristina, Gianniantonio Domina, Emanuele Fanfarillo, Simonetta Fascetti, Tiberio Fiaschi, Gabriele Galasso, Francesco Mascia, Giuliana Mazzacuva, Giacomo Mei, Pietro Minissale, Riccardo Motti, Enrico Vito Perrino, Rosa Maria Picone, Lorenzo Pinzani, Lina Podda, Giovanna Potenza, Leonardo Rosati, Adriano Stinca, Gianmarco Tavilla, Clizia Villano, Robert Philipp Wagensommer, Giovanni Spampinato

**Affiliations:** 1 AGRARIA Department, Mediterranean University of Reggio Calabria, Loc. Feo di Vito snc, 89122 Reggio Calabria, Italy; albertocap1792@gmail.com (A.C.); giuseppe.caruso@unirc.it (G.C.); giulianamazzacuva@gmail.com (G.M.); gspampinato@unirc.it (G.S.); 2 Department of Life Sciences, University of Siena, Via P.A. Mattioli 4, 53100 Siena, Italy; claudia.angiolini@unisi.it (C.A.); emanuele.fanfarillo@unisi.it (E.F.); tiberio.fiaschi2@unisi.it (T.F.); fr.maxia@gmail.com (F.M.); 3 National Biodiversity Future Center (NBFC), Piazza Marina 61, 90133 Palermo, Italy; giulio.barone01@unipa.it (G.B.); lorenzo.pinzani@uniroma3.it (L.P.); 4 Centre for Conservation of Biodiversity (CCB), Department of Life and Environmental Sciences, University of Cagliari, Viale Sant’Ignazio da Laconi 13, 09123 Cagliari, Italy; bacchet@unica.it (G.B.); giacomo.calvia@gmail.com (G.C.); lina.podda@unica.it (L.P.); 5 PLANTA/Center for Research, Documentation and Training, Via Serraglio Vecchio 28, 90123 Palermo, Italy; bajona@centroplantapalermo.org; 6 Section of Botany, Natural History Museum of Milan, Corso Venezia 55, 20121 Milano, Italy; parajubaea@gmail.com (E.B.); gabriele.galasso@comune.milano.it (G.G.); 7 Department of Agriculture, Food and Forest Science, University of Palermo, Viale delle Scienze Bldg. 4, 90128 Palermo, Italy; emilio.digristina@unipa.it (E.D.G.); gianniantonio.domina@unipa.it (G.D.); 8 Department of Agricultural, Food and Environmental Sciences (D3A), Marche Polytechnic University, 60131 Ancona, Italy; nellobisco@gmail.com (N.B.); bonsantodaniele@gmail.com (D.B.); 9 Faculty of Agricultural, Environmental and Food Sciences, Free University of Bolzano, Piazza Università 5, 39100 Bolzano, Italy; giacomo.mei@unibz.it; 10 Department of Biological, Geological and Environmental Sciences, University of Catania, Via A. Longo 19, 95125 Catania, Italy; cambria_salvatore@yahoo.it (S.C.); p.minissale@unict.it (P.M.); 11 Istituto Tecnico Agrario “V. Emanuele II”, Via Cortese 1, 88100 Catanzaro, Italy; 12 Department of ChiBioFarAm, University of Messina, 98166 Messina, Italy; crisafullia@unime.it (A.C.); rpicone@unime.it (R.M.P.); 13 Department of Biology, University of Naples Federico II, Botanical Garden, Via Foria 223, 80139 Naples, Italy; emanuele.delguacchio@unina.it; 14 School of Agriculture, Forestry, Food and Environment, University of Basilicata, Via Ateneo Lucano 10, 85100 Potenza, Italy; simonetta.fascetti@unibas.it (S.F.); giao76.potenza@gmail.com (G.P.); leonardo.rosati@unibas.it (L.R.); 15 Department of Pure and Applied Sciences (DiSPeA), University of Urbino “Carlo Bo”, Via Aurelio Saffi, 2, 61029 Urbino PU, Italy; 16 Department of Agricultural Sciences, University of Naples Federico II, Via Università 100, 80055 Naples, Italy; motti@unina.it (R.M.); clizia.villano@unina.it (C.V.); 17 CIHEAM, Mediterranean Agronomic Institute of Bari, Via Ceglie 9, 70010 Valenzano, Italy; enricoperrino@yahoo.it; 18 Department of Science, University of Roma Tre, Viale G. Marconi, 446, 00146 Rome, Italy; 19 Department of Environmental, Biological and Pharmaceutical Sciences and Technologies, University of Campania Luigi Vanvitelli, 81100 Caserta, Italy; adriano.stinca@unicampania.it; 20 National Research Council of Italy, Institute of Atmospheric Pollution Research (CNR-IIA), c/o Interateneo Physics Department, 70125 Bari, Italy; gtavilla@outlook.com; 21 Faculty of Education, Free University of Bozen-Bolzano, Viale Ratisbona 16, 39042 Brixen-Bressanone, Italy; robertphilipp.wagensommer@unibz.it

**Keywords:** biodiversity, biological invasions, floristic list, herbarium, IAS, Italian regions, species distribution

## Abstract

Despite the wide amount of scientific contributions published on alien plant species, their diffusion dynamics, and their interactions with native taxa, it is increasingly difficult to slow down their spreading and their negative impact on habitats. Last recent years, in fact, a sharp rise in the number of new alien plant taxa introduced in Italy and Europe has been recorded. The aim of this work is to investigate most of the Italian territory in order to verify whether this alarming trend is still underway. Specimen collections and/or observations of alien plants have been performed in as many as 12 Italian regions. All the collected specimens are stored in public or private herbaria. Taxa have been identified according to the literature from the countries of origin of the investigated taxa, while the nomenclature followed the current international references. Updates on 106 taxa are reported. In particular, among 117 new records, 89 are first records, 27 are changes to status and there is 1 extinction. Seven new taxa for Italian alien flora are reported, two of which are new to Europe. The administrative regions with the highest number of records are Calabria (48), Sardegna (17) and Sicilia (15). Five of the surveyed taxa, for the first time, have been considered invasive aliens to Italian territory. The unfrequent amount of original results provided by this work, over the simple importance of data itself, proves how floristic investigation, still today, represents one of the most effective tools in broadening the current knowledge about alien taxa and their dynamics.

## 1. Introduction

The issue of alien plant invasions, while slowly gaining increasing global resonance, already has multiple significant impacts on ecosystems and mankind’s activities [1,2]. One crucial aspect of this worldwide phenomenon is represented by the correct identification and mapping of alien species occurring in each specific geographic area. Recent research has contributed to a better understanding of the dynamics of alien plant spreading [3], but during recent years, there has been a remarkable growth in studies on alien flora in many countries being performed, even those where, until now, little attention has been paid to this issue with possible negative consequences [4,5]. The most recent scientific literature [6] emphasized the urgency of understanding not merely the occurrence of alien plant taxa, but also the possible interactions with native flora. The integrated approach to alien plant taxa management, also highlighted by recent contributions on alien flora in Calabria [7], reveals the need for evidence-based strategies to mitigate negative impacts and promote sustainable coexistence between native and allochthonous species.

Although Mediterranean basin habitats are considered quite resistant to plant species invasion [8], the phenomenon is, however, gradually increasing [9]. While a general pattern in the features of alien flora invading climatically similar regions is lacking, because largely they are dependent on local conditions [10], it is clear that climate change and invasive alien species increasingly threaten the Mediterranean seagrass communities [11]. In freshwater ecosystems, native species proved to be more susceptible to increased invasion of submerged macrophytes and eutrophication [12]. However, invasive alien plant taxa also establish in almost all types of terrestrial environments [13], often determining important ecosystem modification [14,15,16]. Although not all introduced plants become invasive [17,18], the ability to become invasive should not be underestimated, both for casual and naturalized alien taxa [19]. Alien taxa are often introduced into urban green spaces, having more advantages than disadvantages compared to native species [20].

Many checklists have been recently published worldwide, at regional [21] and national [22] levels, in order to better highlight the diachronic changes locally occurring. Furthermore, in Italy, the knowledge of alien flora in different regions has significantly increased in the last decade [23,24,25,26,27,28,29,30,31,32,33,34,35,36,37,38,39,40], although much attention has been paid to highly prioritized IAS in Italy and other European countries, representing a potential threat to the national flora [41,42].

The overall aim of this study is to expand knowledge of the Italian allochthonous vascular flora. The specific objectives of this research are as follows:

(a) To report new taxa for Italy and Europe.
(b) To update the distribution of already known taxa and their occurrence status at regional, national, or continental levels.
(c) To make a synthesis of the current knowledge on allochthonous flora of Italy.
(d) To analyze the floristic similarities among the 20 administrative regions of Italy regarding allochthonous flora.
(e) To analyze the trend of alien taxa in Italy from 2010 to 2023 and their influence on the overall floristic composition (natives + aliens) in the Italian territory.

## 2. Results and Discussion

The floristic data in the present work are the result of field and herbarium research carried out in twelve Italian regions: Basilicata, Calabria, Campania, Lazio, Liguria, Molise, Puglia, Sardegna, Sicilia, Toscana, Umbria, and Trentino Alto Adige.

In this study, 106 alien taxa are considered (Supplementary Materials Table S1). A summary of the data collected is shown in Table 1. Specifically, 89 first records, 27 changes in status, and 1 extinction are reported for the regions surveyed for a total of 117 new records.

Table 2 shows the invasiveness status of the new alien taxa presented in this work. Most reports concern Calabria, with 39 casual alien, 6 naturalized alien, and 3 invasive alien taxa reported. An important number of taxa has also been contributed by the two main Italian island regions, where 12 casual alien, 1 naturalized alien and 4 invasive alien taxa are reported for Sardegna, and 12 casual alien and 3 naturalized alien taxa for Sicily. It is noteworthy that 11 status changes from naturalized alien to invasive alien are reported for some of the surveyed regions. Five of these taxa are also considered invasive aliens for the first time in Italy: *Leucaena leucocephala* (Lam.) de Wit subsp. *glabrata* (Rose) Zárate (Calabria), *Bidens aurea* (Aiton) Sherff (Sardegna), *Jaborosa integrifolia* Lam. (Sardegna), *Melia azedarach* L. (Calabria), *Polanisia dodecandra* (L.) DC. subsp. *trachysperma* (Torr. & A.Gray) Iltis (Toscana). There are also 23 new records of naturalized alien taxa, including 7 first regional records, while the other 16 are status changes; two of these taxa are considered for the first time as naturalized aliens in Italy: *Lantana montevidensis* (Spreng.) Briq. (Calabria) and *Paspalum exaltatum* J.Presl (Liguria). Also significant is the contingent of casual alien taxa recorded for the various regions surveyed in this study: 81 records attributable to 78 taxa.

### 2.1. Floristic List

A detailed floristic list including all the 106 alien taxa reported in this paper for the 12 Italian regions surveyed follows.

**1.**  ***Acacia dealbata* Link**

Fabaceae—Neophyte—South East Australia and Tasmania—Scapose phanerophyte

*First record for Puglia (naturalized alien)*

*Specimen:* 17 August 2023, Chianche Lisce, Vico del Gargano (Foggia), uncultivated olive grove, 41.904593° N–15.940482° E, 461 m a.s.l., *leg. et det.* D. Bonsanto, N. Biscotti (FI).

Note. Along the Italian Adriatic belt, the species was, until now, reported only for Friuli Venezia Giulia [43]. It is a new alien from Puglia. It was observed as a very dense stand with uneven-aged individuals in an uncultivated olive grove.

**2.**  ***Acalypha australis* L.**

Euphorbiaceae—Neophyte—South East China, Japan and Philippines—Scapose therophyte

*First record for Calabria (casual alien)*

*Change in status for Sicilia: from casual alien to naturalized alien (naturalized alien)*

*Specimina*: 5 May 2020, Via dell’Università, Reggio Calabria, roadside/sidewalk, 38.121260° N–15.661054° E, 73 m a.s.l., *leg. et det.* V.L.A. Laface (REGGIO); 20 July 2021, tra via Vincenzo de Grazia e Piazza Giuseppe Garibaldi, Catanzaro, in a planter, 38.908692° N–16.589143° E, 341 m a.s.l., *leg.* A. Capuano, *det.* A. Capuano, G. Caruso (REGGIO, Herb. Capuano); 9 October 2022, Copanello Lido, Stalettì (Catanzaro), flowerbed along the road close to the beach, 38.7665555° N–16.5668071° E, 4 m a.s.l., *leg.* A. Capuano, *det.* A. Capuano, G. Caruso (REGGIO, Herb. Capuano); 2 November 2022, Corso Giuseppe Mazzini, Catanzaro, pavement crack, 38.9084028° N–16.5904173° E, 343 m a.s.l., *leg.* A. Capuano, *det.* A. Capuano, G. Caruso (REGGIO, Herb. Capuano); 4 January 2023, Via Progresso, Catanzaro Lido, Catanzaro, in a planter, 38.820846° N–16.613908° E, 4 m a.s.l., *leg.* A. Capuano, *det.* A. Capuano, G. Caruso (REGGIO, Herb. Capuano).

*Observata:* 27 June 2023, Pollara, Isola di Salina (Aeolian Islands), Messina, irrigated crop, 38.578118° N–14.807360° E, 62 m a.s.l., *obs.* A. Crisafulli, R.M. Picone; 6 November 2023, strada Statale 113, Barcellona Pozzo di Gotto (Messina), damp uncultivated, 38.159953° N–15.242206° E; 45 m a.s.l., *obs.* A. Crisafulli; 6 November 2023, vivai Ryolo, Barcellona Pozzo di Gotto (Messina), as a weed inside the nursery outside of the pots, 38.161626° N–15.240982° E; 40 m a.s.l., *obs.* A. Crisafulli.

Note. *Acalypha australis* is known as an introduced species in several countries across the world, where it has been found mainly in disturbed and anthropized areas [3,44,45,46,47]. In Italy, it has been reported for several northern Italian regions and for Lazio as a casual alien or naturalized alien [43]. It was recently reported for Sicilia as a casual alien [27]. This species is easily distinguished from the similar Asian taxon *Acalypha indica* L. (which has been erroneously first reported to Italy by Zanotti [48] instead of *A. australis*, as indicated in [27]) by bigger female bracts (more than 0.5 cm) and from the North American taxon *Acalypha virginica* L. by ovate-cordate female bracts with crenate-denticulate margin instead of deeply lobed [45,47,49].

The spread of this species is probably promoted by nursery substrates, from which seeds or small seedlings can be dispersed [50]. In most of the recorded localities plants growing both inside and outside pots hosting ornamentals have been observed. It is possible that this species could spread in the future especially in disturbed sites and green areas.

In Sicilia the species has been found in two Messina stands. First individuals have been observed in a garden on the island of Salina, where it has been traced the nursery origin of the seeds for over three years. Currently, the species behaves like a weed, producing hundreds of specimens. New individuals grow not far away along a roadside in a damp anthropized area.

**3.**  ***Acalypha virginica* L.**

Euphorbiaceae—Neophyte—East Central U.S.A.—Scapose therophyte

*First record for Sardegna (casual alien)*

*Change in status for Campania: from casual alien to naturalized alien (naturalized alien)*

*Specimina:* 22 October 2022, Sa Mitza de su Truncu, Sardara (Sud Sardegna), agricultural and rural habitats: fallow lands on wet and altered soils, rubble, 39.608956° N–08.821064° E, 125 m a.s.l., *leg. et det.* F. Mascia, G. Bacchetta, G. Calvia (CAG); 6 June 2021, Colli Aminei, Napoli, cultivated areas and roadsides, 40.871227° N–14.223320° E, 240 m a.s.l., *leg*.: R. Motti, *det*. R. Motti, C. Villano, AS001 (PORUN).

Note. *Acalypha virginica* is a therophyte native to North America, reported as invasive, naturalized or casual alien in some Italian regions. This species is reported as an invasive alien in Liguria, Lombardia, Veneto and Friuli Venezia Giulia; as a naturalized alien in Piemonte, Trentino Alto Adige, Emilia Romagna, Toscana and Puglia; and as a casual alien in Campania and Calabria [43].

In Campania, this species has been reported only for Avellino, Napoli and Portici [51,52] without any geographical references. Here, we report three established populations observed over the years, located in cultivated areas and roadsides in the Colli Aminei area and Camaldoli hill in the city of Napoli. Based on our observations, we propose the status of “naturalized” in Campania for this species.

In Sardegna, several individuals of this species grow on disturbed soil, roadsides, and dumps, likely introduced through nursery and agricultural activities (e.g., contaminated growing substrates or infested phytocells).

**4.**  ***Achillea filipendulina* Lam.**

Asteraceae—Neophyte—South West Asia—Scapose hemicryptophyte

*First record for Calabria (casual)*

*Specimen:* 8 August 2022, Contrada Gornelle, Reggio Calabria, border of a chestnut forest, 38.144874° N–15.817743° E, 1295 m a.s.l., *leg. et det.* V.L.A. Laface (REGGIO).

Note. The species was found at the side of a mountain road with several individuals renewed since 2020 (V.L.A. Laface personal observation). In Italy, the species is reported in Emilia-Romagna as naturalized, while in Lombardia, Marche, Piemonte, Sardegna, and Veneto as a casual alien [43], and was recently reported for Toscana according to Galasso et al. [39].

**5.**  ***Actinidia deliciosa* (A.Chev.) C.F.Liang & A.R.Ferguson**

Actinidiaceae—Neophyte—China—Climbing phanerophyte

*First record for Calabria (casual alien)*

*Specimen:* 13 May 2023, Via Provinciale, Melia di Scilla (Reggio Calabria), roadside scarp with *Castanea sativa* Mill., 38.237156° N–15.734642° E, 582 m a.s.l., *leg. et det.* V.L.A. Laface, G. Mazzacuva (REGGIO).

Note. *Actinidia deliciosa* is cultivated worldwide for fruit yield [53]. The observed individual grows on a slope climbing on chestnut trees; its origin is unknown, as there are no cultivated plants in the area. In Italy, it is reported to be a casual alien, especially in the northern and central regions bordering the Tyrrhenian Sea [43].

**6.**  ***Aeonium arboreum* (L.) Webb & Berthel.**

Crassulaceae—Archaeophyte—Canary Is., Morocco—Nanophanerophyte

*First record for Puglia (casual alien)*

*Specimen*: 23 February 2022, Peschici (Foggia), at the village entrance, in ledges on vertical rock, 41.919412° N–15.920386° E, 114 m a.s.l., *leg. et det.* D. Bonsanto, N. Biscotti (Herb. Bonsanto).

Note. In Italy, this species is reported especially in the Tyrrhenian regions [43]. It is a new alien for Puglia. Only one specimen was found in a small pocket of soil on a vertical rock face near the town of Peschici, which probably escaped from pot cultivation.

**7.**  ***Agave angustifolia* Haw. subsp. *angustifolia***

Asparagaceae—Neophyte—Central America and Mexico—Rosulate hemicryptophyte

*First record for Calabria (casual alien)*

*Specimen:* 27 June 2022, Pellaro, Reggio Calabria, sandy beach, 38.015869° N–15.635070° E, 4 m a.s.l., *leg. et det.* C.M. Musarella (REGGIO).

*Observata:* 27 June 2022, Pellaro, Reggio Calabria, sandy beach, 38.015420° N–15.635246° E, 4 m a.s.l. *obs.* C.M. Musarella; 10 July 2022, Pellaro, Reggio Calabria, sandy beach, 38.015869° N–15.635070° E, 4 m a.s.l., *obs.* C.M. Musarella; 17 July 2022, Pellaro, Reggio Calabria, sandy beach, 38.015869° N–15.635070° E, 4 m a.s.l., *obs.* C.M. Musarella; 5 August 2022, Pellaro, Reggio Calabria, sandy beach, 38.015869° N–15.635070° E, 4 m a.s.l., *obs.* C.M. Musarella.

Note. The specimen of *Agave angustifolia* subsp. *angustifolia* reported was observed and collected on a sandy beach where several private villas have a garden in front of the sea, outside of which they grow thorny plants to avoid bathers stopping. A coastal storm probably removed an adult individual and relocated him lying a few meters from the shoreline, in a higher position. Here, the plant continued to vegetate until it flowered and bore fruit, without giving rise to new shoots at the base. To date, this species is reported only for Sicilia and Puglia, representing the second record for the Italian peninsula [43].

**8.**  ***Agave attenuata* Salm-Dyck subsp. *attenuata***

Asparagaceae—Neophyte—Mexico—Caespitose phanerophyte

*First record for Calabria (casual alien)*

*Observata:* 20 November 2022, Bocale II, Reggio Calabria, scarp roadside, 37.992759° N–15.648316° E, 22 m a.s.l., *obs. et det.* V.L.A. Laface, C.M. Musarella; 5 May 2021, Catona, Reggio Calabria, roadside, 38.174053° N–15.649314° E, 23 m a.s.l., *obs.* V.L.A. Laface.

Note. The observed individuals grow in the proximity of abusive dumps of inert and garden pruning waste material, probably deriving from waste cuttings from nearby gardens: in fact, *A. attenuata* subsp. *attenuata* is commonly used as an ornamental species. The areas where the observed individuals grow are not easily accessible, so they were probably not planted there, nor currently cultivated. In Italy, it is reported as a naturalized alien in Sardegna and casual alien in Lazio and Sicilia [43].

**9.**  ***Allium cepa* L.** [≡*Porrum cepa* (L.) Rchb.]

Amaryllidaceae—Archaeophyte—Turkmenistan (Culton)—Bulbose geophyte

*First record for Sicilia (casual alien)*

*Specimen:* 25 May 2020, Via Castello di Roccella, Campofelice di Roccella (Palermo), roadside, 37.999087° N–13.884798° E, 5 m a.s.l., *leg.* G. Domina, *det.* G. Domina, E. Di Gristina, G. Barone (SAF100131).

Note. Culton was domesticated from *Allium vavilovii* Popov & Vved., and is widely used as a food plant. Two individuals have been found along a road, probably coming from nearby cultivated plants. *Allium cepa* occurs throughout Italy as a casual alien and is unreported only in Valle d’Aosta, Trentino-Alto Adige, Liguria and Puglia [43].

**10.**  ***Allium tuberosum* Rottler ex Spreng.**

Amaryllidaceae—Neophyte—East Asia—Bulbose geophyte

*First record for Sardegna (casual alien)*

*Specimen:* 15 April 2023, Valle di Palabanda, Cagliari, synanthropic habitats, 39.223038° N–09.112260° E, 38 m a.s.l., *leg. et det.* F. Mascia, G. Calvia, G. Bacchetta (CAG).

Note. Several spontaneously growing individuals were found in the Palabanda valley (Hortus Botanicus Karalitanus, Cagliari), likely introduced through weed seeds present in the topsoil. This species has previously been reported for several Italian regions as a naturalized alien in Emilia-Romagna, Marche, Toscana, Veneto, Umbria and as a casual alien in Friuli Venezia Giulia, Lazio, Lombardia, Trentino-Alto Adige and Sicilia [43].

**11.**  ***Amaranthus emarginatus* Salzm. ex Uline & W.L.Bray subsp. *emarginatus*** [≡*A. blitum* L. subsp. *emarginatus* (Salzm. ex Uline & W.L.Bray) Carretero, Muñoz Garm. & J.Pedrol]

Amaranthaceae—Neophyte—South America—Scapose therophyte

*Change in status for Campania: from casual alien to naturalized alien (naturalized alien)*

*Specimen:* 4 October 2023, Trincerone (via Pietro da Eboli), Salerno, pavings, 40.679395° N–14.767170° E, 30 m a.s.l., *leg. et det.* E. Del Guacchio, *Herb. Del Guacchio n. 7851* (NAP, barcode NAP0002648).

Note. Reported for several Italian regions as a casual alien or naturalized alien [43], *A. emarginatus* subsp. *emarginatus* is rare or more probably overlooked in Campania [51]. These authors reported a specimen from the same population indicated here: its self-replacement by seeds for more than ten years allows us to regard this taxon as a naturalized alien in Campania.

**12.**  ***Amorpha fruticosa* L.**

Fabaceae—Neophyte—North America—Caespitose phanerophyte

*Change in status for Umbria: from naturalized alien to invasive alien (invasive alien)*

*Specimen:* 30 August 2022, Ciconia, Orvieto (Terni), riparian vegetation, 42.724275° N–12.140929° E, 108 m a.s.l., *leg. et det.* T. Fiaschi, C. Angiolini (SIENA).

Note. Abundant populations of *Amorpha fruticosa* were found on the Tiber river, colonizing gravel beds. This species is recorded as an invasive alien in most of north and central Italy, and it is naturalized alien in all of the Italian regions except Valle d’Aosta, where it is a casual alien [43].

**13.**  ***Annona cherimola* Mill.**

Annonaceae—Neophyte—South America—Scapose phanerophyte

*First record for Sicilia (casual alien)*

*Specimen:* 9 August 2023, Contrada Solfarelli, Campofelice di Roccella (Palermo), *Citrus* grove, 37.992941° N–13.880864° E, 10 m a.s.l., *leg.* G. Domina, *det.* G. Domina, E. Di Gristina, G. Barone (SAF100133).

Note. *Annona cherimola* is a food plant. Several individuals of different ages have been found in an irrigated *Citrus* groove nearby adult fruiting plants. The species is present in Italy as a casual alien only in Calabria [43].

**14.**  ***Anredera cordifolia* (Ten.) Steenis**

Basellaceae—Neophyte—South America—Rhizomatose geophyte

*Change in status for Campania: from naturalized alien to invasive alien (invasive alien)*

*Specimen:* 16 October 2023, Ischia Porto, via Baldassarre Cossa, Ischia (Napoli), hedges, 40.741746° N–13.936464° E, 12 m a.s.l., *leg.* R. Vallariello, R. Scotti, *det.* E. Del Guacchio, *Herb. Del Guacchio n. 7852* (NAP, barcode NAP0002649).

Note. The new locality is the first for Ischia island, where *A. cordifolia* is to be regarded as a casual alien by now. Nevertheless, this finding confirms the invasiveness of the plant, apparently relying only on vegetative propagules in southern Italy [54]. For the introduction and spontaneous diffusion of this species in Campania, as well as its diffusion throughout the mild zones of the region, see [51]. Due to its rapidity of diffusion, the species fully meets the definition of invasive alien species proposed by Pyšek et al. [55]. Furthermore, the local impact of *A. cordifolia*, especially on native vegetation, is impressive, especially when it covers trees with its rapid growth [56]. For these reasons, we believe that the invasive status is more appropriate for this species, as already proposed for other Italian regions (Toscana, Sicilia) [43].

**15.**  ***Araujia sericifera* Brot.**

Apocynaceae—Neophyte—South America—Climbing phanerophyte

*First record for Basilicata (casual alien)*

*Change in status for Calabria: from casual alien to naturalized alien (naturalized alien)*

*Specimina:* 10 July 2023, old town of Scalea (Cosenza), roadside rainwater drainage, 39.818145° N–15.791227° E, 59 m a.s.l., *leg. et det.* Fascetti S., Potenza G., Rosati L. (HLUC); 20 July 2023, Via Fiumicello, S. Maria del Cedro (Cosenza), water drainage ditch at the edge of the vegetable gardens, 39.742617° N–15.81662° E, 5 m a.s.l., *leg. et det.* Fascetti S., Potenza G., Rosati L. (HLUC); 2 July 2023, old town of Maratea (Potenza), roadside rainwater drainage, 39.992612° N–15.720666° E, 317 m a.s.l., *leg. et det.* Fascetti S., Potenza G., Rosati L. (HLUC).

Note. *Araujia sericifera* is a liana native to central-east South America. The species has been reported in the flora of almost all Italian regions as a casual or naturalized alien [43] and in recent years it showed a rapid expansion moving from the status of casual alien to naturalized or invasive alien (e.g., Lazio, Campania) [57,58]. In the regions reported here, the species was introduced about 10 years ago as ornamental. The Basilicata record fills the distribution gap of this taxon with the neighboring regions [57,59,60].

**16.**  ***Asparagus setaceus* (Kunth) Jessop**

Asparagaceae—Neophyte—East South Africa—Climbing phanerophyte

*First record for Basilicata (casual alien)*

*Specimen:* 10 May 2023, Campus Universitario, Potenza, abandoned orchard, 40.647515° N–15.807614° E, 710 m a.s.l., *leg. et det.* Fascetti S., Potenza G., Rosati L. (HLUC).

Note. *Asparagus setaceus* is commonly used as an ornamental plant. It spreads in wild environments having probably escaped cultivation in surrounding gardens. In the above localities, it was found in an abandoned orchard. It is present in southern Italian regions as a naturalized alien in Calabria [58], Campania and Sicilia, whereas it is a casual alien in Lazio, Puglia, Sardegna, Trentino-Alto Adige [43].

**17.**  ***Bauhinia purpurea* L.** [≡*Phanera purpurea* (L.) Benth.]

Fabaceae—Neophyte—South Asia—Scapose phanerophyte

*First record for Italy (casual alien) and Europe*

*Specimen:* 9 August 2023, Via Salvatore Spinuzza, Palermo, interstices of the sidewalks and pots, 38.121363° N–13.359845° E, 30 m a.s.l., *leg.* E. Di Gristina, *det.* E. Di Gristina, G. Domina (SAF100132).

Note. *Bauhinia purpurea* is used for ornamental purposes due both for the attractive flowers and for its decorative foliage. Some individuals have been found in the interstices of the sidewalks and in the pots, probably coming from nearby adult fruiting plants. This is the first record for Italy and the European continent [61]. The native range of the species is the Indian Subcontinent to Myanmar [61]. *Bauhinia purpurea* is reported as introduced in South East Asia, West Australia, Queensland, Iraq, Central and South Africa, Central and South America.

**18.**  ***Bauhinia variegata* L.**

Fabaceae—Neophyte—South Asia—Scapose phanerophyte

*First record for Europe (casual alien)*

*Specimen:* 20 July 2023, Sant’Agata li Battiati (Catania), roadside, 37.56469° N–15.08221° E, 345 m a.s.l., *leg.* S. Cambria, G. Tavilla, *det.* S. Cambria (CAT).

Note. According to POWO, the native range of *Bauhinia variegata* is the Indian Subcontinent to China (S. Yunnan) [61]. While conducting field surveys in Sicilia, several individuals were observed growing along the roadside of urban environments in Catania and Palermo. This represents the first record for Italy and Europe and it is considered as a casual alien species. It is likely that these plants escaped from private gardens. It is commonly grown as an ornamental tree in tropical and subtropical regions. This species has seeds that are dispersed by wind, allowing them to easily escape cultivation and establish in several habitats [62]. Its seeds can remain viable for over a year, making it difficult to manage [63]. Additionally, it has become an invasive alien species in the southern United States, South Africa, Canary Islands, and East Australia [64,65].

**19.**  ***Begonia Semperflorens* Cultorum Group**

Begoniaceae—Neophyte—South America—Bulbose geophyte

*First record for Calabria (casual)*

*Specimen:* 24 August 2023, Zungri (Vibo Valentia), roadside, 38.653036° N–15.985563° E, 541 m a.s.l., *leg. et det.* V.L.A. Laface (REGGIO).

Note. This represents a complex of horticultural hybrids derived by South American species [43]. The observed individual grows between the asphalt and the wall, born from seeds from nearby houses. In Italy, the species is reported as a casual alien only in Campania [66].

**20.**  ***Bidens aurea* (Aiton) Sherff**

Asteraceae—Neophyte—North Central America—Scapose hemicryptophyte

*Extinct for Puglia*

*Change in status for Sardegna and for Italy: from naturalized alien to invasive alien (invasive alien)*

*Specimina:* 27 October 2016, to the west of the town, at the beginning of the road to in Lamis, San Nicandro Garganico (Foggia), roadside, 41.823250° N–15.549489° E, 250 m a.s.l., *leg. et det.* R.P. Wagensommer (FI, Herb. R.P. Wagensommer); 22 July 2023, near the wastewater treatment plant, Sadali, Sud Sardegna (SU), wet meadows and uncultivated fields, 39.816503° N–09.277872° E, 703 m a.s.l., *leg. et det.* F. Mascia, L. Podda, G. Bacchetta (CAG).

*Observatum:* 10 July 2022, Padru ‘èciu, Sardara (Sud Sardegna), roadside and wet uncultivated fields, 39.608914° N–08.821092° E, 125 m a.s.l., *leg. et det.* F. Mascia, L. Podda, G. Bacchetta.

Note. *Bidens aurea* was recently indicated as a naturalized alien in Puglia, based on a report from a site in which it was collected 30 years before (San Nicandro Garganico, presso San Giuseppe) [25]. In 2022, we could not find the species in the same site. Considering that the small occurring site has been transformed by paving works, *B. aurea* must be considered as an extinct alien species in Puglia.

In Sardegna, a population of numerous individuals was found within an area of approximately one hundred square meters (Sadali, Sud Sardegna). Moreover, we also observed several individuals along roadsides and in damp uncultivated fields (Sardara, Sud Sardegna).

**21.**  ***Bothriochloa laguroides* (DC.) Herter**

Poaceae—Neophyte—America—Caespitose hemicryptophyte

*First record for Calabria (casual alien)*

*Specimen:* 18 August 2022, Villa Comunale di Scalea (Cosenza), mowed meadow, 39.814178° N–15.787827° E, 4 m a.s.l., *leg.* A. Stinca, M. Ravo, *det.* A. Stinca, IT 8344.

Note. *Bothriochloa laguroides*, native to America, was reported as a naturalized alien in Liguria and Campania [43], but had never been found in the region before the present record. The collected specimens were growing in a dry mowed meadow characterized by many synanthropic and exotic species, such as *Paspalum notatum* Flüggé.

**22.**  ***Bougainvillea spectabilis* Willd.**

Nyctaginaceae—Neophyte—Brazil—Climbing phanerophyte

*First record for Calabria (casual alien)*

*Specimen:* 27 July 2023, Collina di Pentimele, Reggio Calabria, on the sandy ridges of the hill, 38.130804° N–15.664297° E, 186 m a.s.l., *leg. et det.* V.L.A. Laface (REGGIO).

Note. Some individuals of *Bougainvillea spectabilis* were observed from pruning material from a nearby sports center (shooting range), where the plant grows for ornamental purposes on fence nets. In Italy, it is reported as a casual alien in Abruzzo and Sardegna [43].

**23.**  ***Broussonetia papyrifera* (L.) Vent.**

Moraceae—Neophyte—East South Asia—Caespitose phanerophyte

*Change in status for Toscana: from naturalized to invasive alien (invasive alien)*

*Observatum:* 9 August 2023, Via del Masso, Poggibonsi, Siena, ruderal habitats, 43.464763° N–11.143293° E, 102 m a.s.l., *obs.* T. Fiaschi.

Note. *Broussonetia papyrifera* is widespread in ruderal sites, spreading by vegetative reproduction. Besides Toscana, it was recently considered an invasive alien in the neighboring Lazio region [37]. *Broussonetia papyrifera* occurs in all Italian regions except Valle d’Aosta. The species is an invasive alien in many regions of northern Italy and mainly a naturalized alien in the rest of the country [43].

**24.**  ***Canna glauca* L.**

Cannaceae—Neophyte—America—Rhizomatose geophyte

*First record for Calabria (casual alien)*

*Specimen:* 4 July 2022, Gasponi, Drapia (Vibo Valentia), roadside, 38.662431° N–15.906258° E, 250 m a.s.l., *leg.* G. Mei, F. Di Laura, *det.* G. Mei, A. Stinca (Herbarium Austroitalicum).

Note. Some individuals of different ages and sizes of *C. glauca*, of which only one with flowers and fruit, emerged from the cracks of the road shoulder and the spaces between the prefabricated concrete components of the road ditch immediately before the bridge that connects Gasponi to Drapia. This species is reported only for Emilia Romagna and Sicilia as a casual alien [43]; therefore, this is the second report for the Italian Peninsula and the second for South Italy.

**25.**  ***Catalpa speciosa* Teas**

Bignoniaceae—Neophyte—U.S.A.—Scapose phanerophyte

*First record for Toscana (naturalized alien)*

*Specimen:* 21 June 2019, Selvapiana, Rufina (Firenze), riparian forest, 43.803679° N–11.469294° E, 100 m a.s.l., *leg. et det.* L. Pinzani (FI).

Note. *Catalpa speciosa* is an ornamental species native to North America. In Italy, it has been already recorded as naturalized in Piemonte and as a casual alien in Lombardia [43]. In the surveyed site, the species forms a small population with mature trees already present at the time of the finding (2019) along the left bank of the Sieve River. Over 4 years of observation, the species has shown a gradual trend of expansion, and a few isolated specimens are already spreading in a nearby *Quercus cerris* L. woodland.

**26.**  ***Cedrus atlantica* (Endl.) G.Manetti ex Carrière**

Pinaceae—Neophyte—Algeria and Morocco—Scapose phanerophyte

*Change in status for Toscana: from casual alien to naturalized alien (naturalized alien)*

*Observatum:* 29 May 2021, Monte Luco, Gaiole in Chianti, Siena, artificial plantations, 43.440859° N–11.505792° E, 745 m a.s.l., *obs.* T. Fiaschi, E. Fanfarillo.

Note. Under a plantation established for reforestation purposes, abundant juveniles of *C. atlantica* up to 3 m tall have been found, suggesting that the species is naturalized in the area. The taxon is recorded in most Italian regions, but until now, it was considered naturalized alien only in Sardegna [43].

**27.**  ***Cedrus deodara* (Roxb.) G.Don**

Pinaceae—Neophyte—Central Asia—Scapose phanerophyte

*First record for Lazio (casual alien)*

*Specimina:* 16 February 2020, near casa di preghiera S. Luca, Guarcino, Frosinone, along a pathway in the wood, 41.806101° N–13.324319° E, 737 m a.s.l., *leg. et det.* E. Fanfarillo (SIENA); 7 August 2023, near casa di preghiera S. Luca, Guarcino, Frosinone, along a pathway in the wood, 41.806101° N–13.324319° E, 737 m a.s.l., *leg. et det.* E. Fanfarillo (SIENA).

Note. One young individual of *C. deodara*, approximately 2m tall, was found along a pathway in a deciduous wood. *Cedrus deodara* was until now recorded as a casual alien in several regions of north and peninsular Italy and in Sardegna, while it is a naturalized alien in Lombardia [43].

**28.**  ***Cenchrus longispinus* (Hack.) Fernald**

Poaceae—Neophyte—Central and North America—Scapose therophyte

*First record for Calabria (invasive alien)*

*Specimina:* 30 September 2023, Spiaggia di Praia a Mare, Praia a Mare (Cosenza), sandy beach dunes, 39.880544° N–15.785276° E, 3 m a.s.l., *leg. et det.* V.L.A. Laface (REGGIO); 30 September 2023, Spiaggia di Praia a Mare, Praia a Mare (Cosenza), sandy beach dunes, 39.882124° N–15.785047° E, 4 m a.s.l., *leg. et det.* V.L.A. Laface (REGGIO); 30 September 2023, Spiaggia di Praia a Mare, Praia a Mare (Cosenza), sandy beach dunes, 39.886534° N–15.783776° E, 5 m a.s.l., *leg. et det.* V.L.A. Laface (REGGIO).

Note. *Cenchrus longispinus* has been observed on the sandy dunes of the Praia a Mare, on the Thyrrenian side of the Cosenza province. It displays invasive behavior, occupying not only all the dunes for many kilometers, but also the flower beds and pavements nearby. The fruits have hooks that allow them to cling easily and firmly to animals and fabrics, but also to the skin itself, which allows them to be carried unintentionally for very long distances. It is reported throughout Italy as invasive alien except in Sardegna, Umbria, Trentino, Valle d’Aosta and Calabria [43].

**29.**  ***Cenchrus purpurascens* Thunb.** [≡*Cenchrus compressus* (R.Br.) Morrone; *Panicum alopecuroides* L.]

Poaceae—Neophyte—East Asia, Indonesia, Malaysia and Australia—Caespitose hemicryptophyte

*First record for Campania (casual alien)*

*Specimen:* 4 October 2023, Viale Unità d’Italia, Salerno, lawns, 40.677163° N–14.775137° E, 15 m a.s.l., *leg. et det.* E. Del Guacchio, *Herb. Del Guacchio n. 7854* (NAP, barcode NAP0002650).

Note. Recently introduced as an ornamental grass, *C. purpurascens* escapes from flowerbeds and colonizes lawns and paving cracks. It spreads by means of hypogeal stolons and seeds. This species has been identified using the taxonomic key reported in [67]. Until today, this species has been known only for a few central-north Italian regions [43]: therefore, this is the first record for Campania and South Italy.

**30.**  ***Chamaecyparis lawsoniana* (A.Murray) Parl.**

Cupressaceae—Neophyte—California and Oregon—Scapose phanerophyte

*First record for Calabria (casual alien)*

*Specimen:* 14 August 2023, Gambarie, Santo Stefano in Aspromonte (Reggio Calabria), in a crack of a fence wall, 38.167825° N–15.834611° E, 1310 m a.s.l., *leg. et det.* C.M. Musarella (REGGIO).

*Observatum:* 14 August 2023, Gambarie, Santo Stefano in Aspromonte (Reggio Calabria), in a crack of a fence wall, 38.167374° N–15.835001° E, 1314 m a.s.l., *leg. et det.* C.M. Musarella.

Note. *Chamaecyparis lawsoniana* is a species native to some states of the United States [61], cultivated for ornamental and reforestation purposes in Italy, where it is reported as a casual alien in various regions of the central-north peninsula and in Sardegna [43]. Both specimens reported here grow in the cracks of various fence walls surrounding private villas where this plant is mainly grown for hedges. This is the first report for Calabria and South Italy.

**31.**  ***Cicer arietinum* L. subsp. *arietinum***

Fabaceae—Archaeophyte—Iran, Iraq and Turkey (Culton)—Scapose therophyte

*First record for Sicilia (casual alien)*

*Specimen:* 18 April 2018, Contrada Giuranella, Agrigento, uncultivated land, 37.345555° N–13.543885° E, 240 m a.s.l., *leg.* G. Domina, *det.* G. Domina, E. Di Gristina, G. Barone (SAF100129).

Note. Culton domesticated from *C. arietinum* subsp. *reticulatum* (Ladiz.) Moreno & Cubero ex Del Guacchio & P.Caputo, widely used as a food plant. Some individuals have been found in uncultivated land, where the plant was presumably grown in previous years. *Cicer arietinum* subsp. *reticulatum* is present throughout Italy as a casual alien and not reported only for Valle d’Aosta, Liguria, Basilicata and Calabria [43].

**32.**  ***Citrullus amarus* Schrad.**

Cucurbitaceae—Neophyte—South Africa—Scapose therophyte

*First record for Italy (casual alien)*

*Specimen:* 24 August 2021, Fundali, Siddi (Sud Sardegna), agricultural and rural habitats, 39.669803° N–08.894621° E, 165 m a.s.l., *leg. et det.* F. Mascia, G. Calvia, G. Bacchetta (CAG).

Note. Some individuals of *Citrullus amarus* were discovered in abandoned fields in Siddi. In Sardegna, this species was likely extensively cultivated in the past for traditional food and medicinal purposes (e.g., *sub Zidra* [68]). *Citrullus amarus* is native to South Africa and is widely cultivated throughout the Mediterranean region, particularly in North African Mediterranean countries [69].

**33.**  ***Citrus ×limon* (L.) Osbeck**

Rutaceae—Archaeophyte—China—Scapose phanerophyte

*First record for Puglia (casual alien)*

*Observatum:* 26 April 2020, Paura, Polignano a Mare (Bari), shrub vegetation, 40.998418° N–17.212255° E, 6 m a.s.l., *obs. et det.* E.V. Perrino.

Note. *Citrus ×limon* is a cultigen derived from artificial hybrids: the hybrid formula is *C. maxima × C. medica × C. reticulata*. It is a tree and grows primarily in the subtropical climatic belt. It is used to treat unspecified medicinal disorders, has environmental uses, as animal food and a medicine and for fuel and food [61]. The individual observed is in a good state of conservation, without particular pathologies, it is tall about 3 m and in the future, it could suffer the probable closure of the sclerophyllous vegetation. In Italy, *Citrus ×limon* occurs only in Sardegna and Campania as a casual alien [43].

**34.**  ***Clerodendrum trichotomum* Thunb.**

Lamiaceae—Neophyte—South East Asia—Scapose phanerophyte

*First record for Calabria (casual alien)*

*Specimen:* 16 August 2023, Caredia-Lacco Strada Provinciale 3, Reggio Calabria, 37.955560° N–15.796405° E, 125 m a.s.l., *leg.* C.M. Musarella, *det*. V.L.A. Laface, C.M. Musarella, G. Spampinato (REGGIO).

Note. Although *C. trichotomum* is widely used and studied for its numerous medicinal properties [70,71,72], in Italy, it has been introduced and is used for ornamental purposes. Already reported as a casual alien in several Italian regions [43], the collected specimen was found together with other well-developed and vigorous plants, probably originating from an older individual along the SP3 Provincial Road.

**35.**  ***Coleus scutellarioides* (L.) Benth.** [≡*Calchas scutellarioides* (L.) P.V.Heath; *Majana scutellariodes* (L.) Kuntze; *Ocimum scutellarioides* L.; *Solenostemon scutellarioides* (L.) Codd]

Lamiaceae—Neophyte—East Asia, Indonesia, Malaysia and Australia (Culton)—Scapose hemicryptophyte

*First record for Basilicata (casual alien)*

*First record for Sicilia (casual alien)*

*Specimina:* 2 June 2023, old town of Maratea, Maratea, Potenza, roadside rainwater drainage, 39.992612° N–15.720666° E, 317 m a.s.l., *leg. et det.* Fascetti S., Potenza G., Rosati L. (HLUC); 23 February 2023, Via Archirafi, Palermo, base of a wall, 38.112358° N–13.371282° E, 20 m a.s.l., *leg. et det.* G. Domina, A. Stinca, G. Barone (SAF100126).

Note. *Coleus scutellarioides* is a culton originated from plants introduced from South East Asia, where it is widely and traditionally used for medicinal purposes and for ornamental purposes due to its highly decorative variegated and multicolored leaves [61]. The species was first introduced into Europe from Java in 1851 by a Dutch horticulturist. The diffusion as an ornamental plant increased considerably in the 1980s following the selection of cultivars with very colored and variously shaped leaves. It was reported as a casual alien both in Lombardia [73] and more recently in Calabria [58]. Some plants, probably spread by seeds from urban green flowerbeds, were observed in the old town of Maratea (Basilicata).

In Sicilia, one individual was found at the base of a wall, probably coming from nearby adult fruiting plants.

**36.**  ***Cucumis melo* L. subsp. *melo***

Cucurbitaceae—Archaeophyte—Paleotropical—Scapose therophyte

*First record for Calabria (casual alien)*

*Specimina:* 3 October 2022, Lungomare Italo Falcomatà, Reggio Calabria, on the beach at the base of seafront’s wall, 38.110069° N–15.642998° E, 1 m a.s.l., *leg.* A. Capuano, *det.* A. Capuano, G. Caruso (REGGIO, Herb. Capuano); 7 September 2022, Via dei Tre Mulini, Reggio Calabria, coming out of a rainwater drainage grate, 38.119686° N–15.662074° E, 68 m a.s.l., *leg.* C.M. Musarella, *det*. V.L.A. Laface, C.M. Musarella, G. Spampinato (REGGIO).

Note. *Cucumis melo* is considered one of the most important horticultural crops in the world. It is probably native to Asia [74] or Africa [75]. *Cucumis melo* shows a good resistance to variable soil saline concentrations and it is often grown along the Mediterranean coasts due to the warm climate [76]. A small group of flowering plants has been observed on a sandy beach a few meters from the sea, along with halophilous native species such as *Cakile maritima* Scop. subsp. *maritima*, *Crithmum maritimum* L., *Salsola* sp. and the alien *Solanum lycopersicum* L. In the first stand, seeds could have been probably dispersed through food wastes. In the second stand, a single plant emerged from a rainwater drainage grate, probably as a result of seed dispersal from the balconies of various apartments occurring along the street.

**37.**  ***Cycas revoluta* Thunb.**

Cycadaceae—Neophyte—China, Japan and Taiwan—Scapose phanerophyte

*First record for Calabria and for South Italy (casual alien)*

*Specimen:* 14 August 2023, Reggio Calabria, inside a water drainage channel along the A2 motorway junction, towards Salerno, 38.125055° N–15.653074° E, 13 m a.s.l., *leg. et det.* C.M. Musarella (REGGIO).

Note. *Cycas revoluta* is an ornamental tree cultivated both in pots or in the field. Only one individual was found with just a leaf sprouted under a water tank abandoned in a water drainage channel. The emerging leaf was collected with some difficulty, but easily allowed taxon recognition. The young individual probably formed from an adventitious shoot thrown from one of the many apartments that line the motorway junction and on whose balconies and terraces various adult plants in pots are grown. *Cycas revoluta* was recently recorded for the first time for Italy as a casual alien [77]: this is the second record for the Italian Peninsula and the first for South Italy and for Calabria.

**38.**  ***Cytisus proliferus* L.f. subsp. *proliferus*** [≡*Chamaecytisus proliferus* (L.f.) Link subsp. *proliferus*]

Fabaceae—Neophyte—Canary Islands—Caespitose phanerophyte

*First record for Italy (casual alien)*

*Specimen:* 2 June 2022, Tanca di Nissa, Capoterra (Cagliari), uncultivated fields and side of paths, 39.161539° N–09.003089° E, 4 m a.s.l., *leg. et det.* F. Mascia, L. Podda, G. Bacchetta (CAG).

Note. Dozens of individuals of *C. proliferus* subsp. *proliferus*, varying in age, have been found in uncultivated areas and trail margins, often in association with *Medicago arborea* L. It is hypothesized that they were historically introduced as fodder plants. Notably, experimental grasslands were established at the discovery site since the early decades of the 19th century [78]. While experimental cultivation of *C. proliferus* subsp. *proliferus* for forage purposes was documented in Lipari [79], there was no evidence of successful reproduction and naturalization. The taxon, originally from the Canary Islands [80], was not documented in previous scientific works across continental Europe and Mediterranean islands [55]. On the contrary, *Cytisus proliferus* is introduced into Australia, California, East Tropical Africa, Hawaii, India, Jawa, and New Zealand [61], and it is considered an invasive alien plant in California, Australia, and New Zealand [81].

**39.**  ***Dahlia imperialis* Roezl ex Ortgies**

Asteraceae—Neophyte—Mexico, Central America and Colombia—Caespitose phanerophyte

*First record for Calabria (casual alien)*

*Specimen:* 25 July 2022, Diminniti, Reggio Calabria, at the border of a chestnut grove, 38.165821° N–15.713256° E, 616 m a.s.l., *leg. et det.* V.L.A. Laface (REGGIO).

Note. *Dahlia imperialis* is a native American species cultivated in much of the world for ornamental purposes [61]. The individual appears as a small tree with many suckers; numerous seedlings have been observed in the nearby area. *Dahlia imperialis* is present in Italy as a casual alien, exclusively in Liguria [43].

**40.**  ***Danaë racemosa* (L.) Moench**

Asparagaceae—Neophyte—West Asia—Rhizomatose geophyte

*First record for Sicilia (casual alien)*

*Specimen:* 20 July 2023, Sant’Agata li Battiati (Catania), roadside, 37.56469° N–15.08221° E, 345 m a.s.l., *leg.* S. Cambria, G. Tavilla, *det.* S. Cambria (CAT).

Note. According to the [43], *D. racemosa* occurs as a casual alien species in Emilia-Romagna, Friuli Venezia Giulia, Lazio, Liguria, Lombardia, Trentino-Alto Adige, Toscana, and Veneto. During fieldwork in Sicilia, it was observed in the territory of Sant’Agata li Battiati (Catania) along the roadside. This is the first record of the species being classified as a causal alien species for Sicilia.

**41.**  ***Diospyros lotus* L.**

Ebenaceae—Neophyte—Asia—Scapose phanerophyte

*First record for Sicilia (casual alien)*

*Specimen:* 23 September 2023, Contrada Nespola, Milo (Catania), *Quercus congesta* and *Acer obtusatum* forest, 37.725839° N–15.112519° E, 765 m a.s.l., *leg. et det.* Minissale (CAT).

Note. *Diospyros lotus* was until now known as naturalized alien throughout north and central Italy [43], while it is missing in the more southern regions and islands, probably also due to the less favorable climatic conditions. The discovery in Sicilia concerns a single specimen on the edge of a dense forest dominated by *Quercus congesta* C.Presl (=*Quercus pubescens* Willd. subsp. *pubescens*) and *Acer obtusatum* Waldst. & Kit. ex Willd. subsp. *aetnense* (Tineo ex Strobl) C. Brullo & Brullo [=*Acer opalus* Mill. subsp. *obtusatum* (Waldst. & Kit. ex Willd.) Gams)]. It is a moderately sized individual capable of bearing fruit.

**42.**  ***Diospyros virginiana* L.**

Ebenaceae—Neophyte—East Central U.S.A.—Scapose phanerophyte

*First record for Sicilia (naturalized alien)*

*Specimina:* 24 April 2018, Contrada Telegrafo Vecchio, Catania (Catania), nitrophilous grassland, 37.502458° N–15.039219° E, 155 m a.s.l., *leg. et det.* P. Minissale (CAT); 21 September 2023, Lungo Viale Marco Polo, San Giovanni La Punta (Catania), roadside/sidewalk, 37.576631° N–15.103428° E, 335 m a.s.l., *leg. et det.* P. Minissale (CAT); 19 April 2023, Contrada Licciardello, Acireale (Catania), nitrophilous ruderal vegetation, 37.616178° N–15.151703° E, 218 m a.s.l., *leg. et det.* P. Minissale (CAT); 19 August 2023, Caselle, Milo (Catania), at the edge of cultivated area, 37.720311° N–15.104775° E, 820 m a.s.l., *leg. et det.* P. Minissale (CAT); 21 September 2023, Macchia di Giarre lungo strada regionale n.75, Giarre (Catania), roadside/sidewalk, 37.711786° N–15.156103° E, 230 m a.s.l., *leg. et det.* P. Minissale (CAT); 24 September 2023, Poggiofelice, Zafferana Etnea (Catania), nitrophilous ruderal vegetation, 37.668047° N–15.099144° E, 550 m a.s.l., *leg. et det.* P. Minissale (CAT).

Note. *Diospyros virginiana* has previously been reported only once for Italy by [27] who reported it for Elba island (Toscana), where the species is naturalized. *Diospyros virginiana* in Sicilia has been observed in various locations on Etna and on the south-west outskirts of Catania where it sometimes forms small but dense populations in habitats such as abandoned crop areas, edges of cultivated fields, roadsides, demonstrating a good propagation capacity from root shoots and perhaps from seeds. The species bears fruit regularly and was probably introduced as a rootstock for *Diospyros kaki* Thunb. Regarding the *Diospyros* genus in Sicilia, Spadaro and Raimondo [82] reported a case of spontaneization of *D. kaki* in the province of Agrigento.

**43.**  ***Drosanthemum floribundum* (Haw.) Schwantes**

Aizoaceae—Neophyte—Cape Provinces—Succulent chamaephyte

*First record for Calabria (casual alien)*

*Specimen:* 25 May 2023, Via Caserma, Reggio Calabria, stone boundary wall, 38.118485° N–15.660423° E, 62 m a.s.l., *leg. et det.* V.L.A. Laface (REGGIO).

Note. *Drosanthemum floribundum* is an ornamental species native to the Cape Province, expanding itself in many countries due to its use in gardening, vigorous flowering, groundcover character and replacement of *Carpobrotus* sp. [83]. It easily reproduces vegetatively, producing long stolons [84,85], hence the vernacular name ‘Jupiter’s beard’ [86], which produce adventitious roots at each internode, easily generating new individuals. The individual observed probably came from some plants grown as ornamentals hanging from the balconies above.

**44.**  ***Dysphania pumilio* (R. Br.) Mosyakin & Clemants** [≡*Chenopodium pumilio* R. Br.]

Amaranthaceae—Neophyte—Australia and Tasmania—Scapose therophyte

*First record for Calabria (casual alien)*

*Specimen:* 7 October 2022, Feo di Vito, Reggio Calabria, near the AGRARIA Department, patch of *Ampelodesmos mauritanicus* garigue by the roadside, 38.120983° N–15.669562° E, 118 m a.s.l., *leg.* A. Capuano, *det.* A. Capuano, G. Caruso (REGGIO, Herb. Capuano).

Note. A population of *Dysphania pumilio* covering a surface exceeding 100 square meters has been found on a patch of grassland, mainly composed by *Ampelodesmos mauritanicus* (Poir.) T.Durand & Schinz and scattered specimens of *Euphorbia rigida* M.Bieb. *Dysphania pumilio*, native to Australia, is known as a noxious weed in several countries all over the world [87,88,89,90,91]. In Italy, it was found for the first time in Milan (Lombardia) in 1939 [92].

**45.**  ***Euphorbia pulcherrima* Willd. ex Klotzsch**

Euphorbiaceae—Neophyte—Mexico and Guatemala—Nanophanerophyte

*First record for Calabria (casual alien)*

*Specimina:* 13 June 2023, Rosalì, Reggio Calabria, citrus grove, bordering the Fiumara wall, 38.201173° N–15.674730° E, 103 m a.s.l., *leg. et det.* V.L.A. Laface (REGGIO); 23 November 2022, Gallico Superiore, Reggio Calabria, citrus grove abandoned for several years, 38.171266° N–15.672696° E, 115 m a.s.l., *leg. et det.* V.L.A. Laface (REGGIO); 20 August 2022, Madonna del Rosario, Archi, Reggio Calabria, unauthorised dumping of waste materials, 38.161348° N–15.662690° E, 42 m a.s.l., *leg. et det.* V.L.A. Laface (REGGIO); 25 January 2022, Sbarre, Reggio Calabria, synanthropic and ruderal vegetation for the recovery of highly altered and disturbed environments, 38.088493° N–15.643763° E, 21 m a.s.l., *leg.* G. Mei, G. Posillipo, *det.* G. Mei (Herbarium Mei).

*Observatum:* 1 January 2023, Pentimele, Reggio Calabria, unauthorized dumping of inert materials, 38.166476° N–15.657660° E, 26 m a.s.l., *obs.* V.L.A. Laface.

Note. The individuals of *Euphorbia pulcherrima* observed are always single and have numerous large suckers, probably from pruning waste from neighboring gardens, where the species is commonly cultivated as an ornamental plant. The species has been observed mainly in areas occupied by landfills, where various types of waste are abandoned, including pruning waste and debris. In Italy, this species is reported as a casual alien, exclusively for Campania and Lazio [93,94].

**46.**  ***Euphorbia serpens* Kunth subsp. serpens**

Euphorbiaceae—Neophyte—America—Reptant therophyte

*First record for Calabria (casual alien)*

*Specimina:* 27 August 2022, in front of the church of San Giuseppe, village of Chianalea, Scilla (Reggio Calabria), in a planter, 38.253789° N–15.722254° E, 4 m a.s.l., *leg.* C. Corazza, A. Capuano, *det.* A. Capuano, G. Caruso (REGGIO, Herb. Capuano); 15 September 2022, via Casalotto, Reggio Calabria, sidewalk curb, 38.123036° N–15.658633° E, 34 m a.s.l., *leg.* A. Capuano, *det.* A. Capuano, G. Caruso (REGGIO, Herb. Capuano); 27 September 2022, viale della Libertà, Reggio Calabria, sidewalk cracks, 38.121203° N–15.654302° E, 23 m a.s.l., *leg.* A. Capuano, *det.* A. Capuano, G. Caruso (REGGIO, Herb. Capuano); 30 September 2022, parking of the Faculty of Engineering, Reggio Calabria, pavement cracks, 38.120300° N–15.666330° E, 81 m a.s.l., *leg.* A. Capuano, *det.* A. Capuano, G. Caruso (REGGIO, Herb. Capuano); 3 October 2022, Feo di Vito, courtyard of the AGRARIA Department, Reggio Calabria, pavement cracks, 38.120776° N–15.668376° E, 138 m a.s.l., *leg.* A. Capuano, *det.* A. Capuano, G. Caruso (REGGIO, Herb. Capuano).

Note. *Euphorbia serpens* has been reported as an introduced weed in disturbed sites in many European countries [95,96,97]. Seeds could have been transported accidentally, maybe with nursery substrates [98]. All the surveyed sites have been observed scattered populations, each composed of a few individuals showing good adaptation to the highly stressful conditions of the urban environment. The collected material has been ascribed to the nominal subspecies (reported for many Italian regions as naturalized alien according to [43]), differently from subsp. *fissistipula* (Thell.) Verloove & Lambinon, on the basis of connate stipules.

**47.**  ***Evansia japonica* (Thunb.) Klatt** [≡*Iris japonica* Thunb.]

Iridaceae—Neophyte—South East Asia—Rhizomatose geophyte

*First record for Calabria (casual alien)*

*Specimen:* 13 May 2023, embankment of the Arvo torrent, Ramundo, San Giovanni in Fiore (Cosenza), humid watershed, 39.260385° N–16.586031° E, 1230 m a.s.l., *leg.* W. Fratto, A. Capuano, G. Caruso, *det.* A. Capuano, G. Caruso (REGGIO, Herb. Capuano).

Note. The Calabrian stand of *E. japonica*, a taxon already known in other Italian regions as a casual alien or naturalized alien [43], could spread in the surroundings due to the availability of similar habitats and affecting native herbaceous riparian and forest communities.

**48.**  ***Fatsia japonica* (Thunb.) Decne. & Planch.**

Araliaceae—Neophyte—Japan and Korea—Caespitose phanerophyte

*First record for Calabria (casual alien)*

*Specimen:* 22 May 2023, Catona, Reggio Calabria, abandoned garden among some *Rubus ulmifolius* plants, 38.188639° N–15.646719° E, 21 m a.s.l., *leg. et det.* V.L.A. Laface (REGGIO).

Note. Asiatic species commonly cultivated as an ornamental, the observed individual of *F. japonica* is probably generated from pruning waste from nearby villas. In Italy, *F. japonica* is reported for Liguria, Sardegna and Toscana as a casual alien [43].

**49.**  ***Fragaria ananassa* Rozier** [≡*Fragaria chiloensis* (L.) Mill. var. *ananassa* (Rozier) Ser.; *Potentilla ananassa* (Rozier) Mabb.]

Rosaceae—Neophyte—North America (Culton)—Reptant hemicryptophyte

*First record for Sicilia (casual alien)*

*Observatum:* 9 June 2023, Mercato delle Pulci, Palermo, interstices of the sidewalks, 38.1147874° N–13.3521088° E, 40 m a.s.l., *obs.* E. Di Gristina.

Note. *Fragaria ananassa* is a fixed hybrid between *F. chiloensis* (L.) Mill. × *F. virginiana* Mill. and it is widely cultivated as a food plant. Some mature individuals have been found in the interstices of the sidewalks, probably coming from nearby adult fruiting plants. *Fragaria ananassa* is recorded in Italy as a casual alien in Piemonte, Lombardia, Veneto, Emilia-Romagna and Marche [43].

**50.**  ***Gaillardia ×grandiflora* Van Houtte**

Asteraceae—Neophyte—Artificial hybrid (Culton)—Scapose hemicryptophyte

*First record for Calabria (casual alien)*

*Specimen:* 13 June 2020, Località Torre, San Ferdinando (Reggio Calabria), in the central cavity of a small concrete road that runs through the houses, 38.496924° N–15.921121° E, 5 m a.s.l., *leg.* S.M. Postiglione, *det.* V.L.A. Laface, C.M. Musarella, G. Spampinato (REGGIO).

Note. *Gaillardia ×grandiflora* has beautiful flowers and for this reason, it is commonly used as an ornamental plant [99] and it is well adapted to Mediterranean climate habitats [100]. The individuals observed are derived from seeds of cultivated plants. In Italy, it is reported in Abruzzo, Emilia-Romagna and Lombardia as a casual alien [43].

**51.**  ***Gazania ×splendens* Hend. & Andr.Hend.**

Asteraceae—Neophyte—Artificial hybrid (Culton)– Scapose hemicryptophyte

*First record for Sardegna (naturalized)*

*Specimen:* 3 January 2023, between Residence Don Diego P. Don Diego ayre, Loiri Porto San Paolo (Sassari), coastal habitats and Mediterranean shrubs, 40.875820° N–09.654050° E, 15 m a.s.l., *leg. et det.* G. Calvia, G. Bacchetta, L. Podda (Herb. Calvia, CAG).

Note. Hundreds of individuals of *Gazania ×splendens* are thriving in grasslands and Mediterranean shrublands along a stretch of coastal countryside within the municipality of Porto San Paolo (north-east Sardegna). These plants may have originated from cultivation at a nearby tourist residence and are rapidly spreading within the natural environments of the surrounding areas. A similar situation has been observed along the south-west coast of Sardegna, specifically in the locality of Su Portu’e Su Trigu (Sant’Anna Arresi, Sud Sardegna province), where the taxon has proliferated in grasslands, scrublands, and sandy and rocky areas near the coast. In this case, as well, it has escaped from neighboring villas.

**52.**  ***Gibasis pellucida* (M.Martens & Galeotti) D.R.Hunt**

Commelinaceae—Neophyte—Central America and Mexico—Scapose therophyte

*First record for Calabria (casual)*

*Specimen:* 18 October 2022, Contrada Lacco, Ortì Inferiore, Reggio Calabria, water drainage, 38.147419° N–15.711679° E, 608 m a.s.l., *leg. et det.* V.L.A. Laface (REGGIO).

Note. According to some scholars (i.e.,: [101]), *G. pellucida* is a hygrophilous species; indeed, the individuals collected grow along a drainage channel of a fountain where the water flows and at the level of the internodes they formed adventitious roots [101]. According to Portal to the Flora of Italy [43], in Italy, this species is reported as a casual alien exclusively in Veneto and hase been mistakenly recorded in Trentino-Alto Adige.

**53.**  ***Glycine max* (L.) Merr. subsp. *max***

Fabaceae—Neophyte—Central and East Asia—Scapose therophyte

*First record for Campania (casual alien)*

*Specimen:* 25 May 2023, Valle d’Ansanto, Benevento, roadside, 40.969739° N–15.104946° E, 450 m a.s.l., *leg.* R. Motti, C. Villano, *det.* R. Motti, C. Villano, AS002 (PORUN).

Note. *Glycine max* (soybean) is a species largely cultivated mainly in central northern Italy. This species is reported as a casual alien in Piemonte, Lombardia, Veneto, Emilia Romagna, Marche and Umbria [43]. Therefore, our recent findings in the Ansanto Valley, in Avellino province, are the first in Campania and concern a few plants spread by seed, probably escaped from cultivation.

**54.**  ***Grevillea robusta* A.Cunn. ex R.Br.**

Proteaceae—Neophyte—East Australia—Scapose phanerophyte

*First record for Calabria (casual alien)*

*Specimen:* 11 November 2021, quartiere Sant’Anna, Reggio Calabria, roadside/sidewalk, 38.101910° N–15.643621° E, 23 m a.s.l., *leg. et det.* V.L.A. Laface (REGGIO).

Note. Numerous seedlings of *G. robusta* were observed along the roadside and on the sidewalks of the city center where several adult plants (3–4 years old) grow in the more open surrounding areas without city cleaning interventions. All the individuals observed derive from seeds of very large plants, used as street trees along the main road. In Italy, it is reported as a casual alien in Campania, Lazio and Sicilia [43].

**55.**  ***Helianthus tuberosus* L.**

Asteraceae—Neophyte—North America—Bulbose geophyte

*Change in status for Sardegna: from naturalized alien to invasive alien (invasive alien)*

*Specimen:* 30 September 2022, Rio Funtanedda, Sceas, Escalaplano (Sud Sardegna), Rivers and streams, 39.608565° N–09.349807° E, 204 m a.s.l., *leg. et det.* L. Podda, F. Mascia, G. Bacchetta (CAG).

Note. *Helianthus tuberosus* is currently invasive along numerous watercourses and channels of Sardegna. Its presence has been observed in various locations, including Arzachena (Sassari), Rio Bucchilalgu [Perfugas (Sassari)]; Bulzi (Sassari), Macomer (Nuoro); Sarcidano [Isili, Serri, Mandas (Sud Sardegna)]; Marmilla; Mandrolisai; and Escalaplano (Sud Sardegna) along Rio Funtanedda for several kilometers.

**56.**  ***Impatiens balsamina* L.**

Balsaminaceae—Neophyte—India and Sri Lanka—Scapose therophyte

*First record for Calabria (casual)*

*Specimina:* 11 October 2021, Ortì Inferiore, Reggio Calabria, water drainage channel at the roadside, 38.145783° N–15.714397° E, 392 m a.s.l., *leg. et det.* V.L.A. Laface (REGGIO); 30 October 2021, Pietrastorta, Reggio Calabria, water drainage channel at the roadside, 38.120976° N–15.690248° E, 634 m a.s.l., *leg. et det.* V.L.A. Laface (REGGIO).

Note. *Impatiens balsamina* is native to South Asia and it is an annual plant commonly known as “garden balsam” or “rose balsam”. It is used in Indian traditional medicine for various diseases and human physiological conditions [102]. In Italy, it is cultivated as an ornamental plant, for its autumn flowering. The plants found in drainage channels originated from seeds coming from nearby villas.

**57.**  ***Isatis tinctoria* L. subsp. *tinctoria***

Brassicaceae—Archaeophyte—East Europe and Turkey—Biennial hemicryptophyte

*Change in status for Puglia: from invasive alien to naturalized alien (naturalized alien)*

*Specimina:* 11 May 2002, at Santuario San Matteo, San Marco in Lamis (Foggia), rocky scrubland, 41.709278° N–15.659162° E, 700 m a.s.l., *leg. et det.* B. Schreiber (MJG); 1 May 2019, west of the town, on the road to San Giovanni Rotondo, Manfredonia (Foggia), roadside, 41.639955° N–15.880871° E, 70 m a.s.l., *leg. et det.* R.P. Wagensommer (Herb. R.P. Wagensommer); 1 May 2019, Posta Padovano, San Giovanni Rotondo (Foggia), roadside 41.712297° N–15.764253° E, 610 m a.s.l., *leg. et det.* R.P. Wagensommer (Herb. R.P. Wagensommer).

*Observata:* 4 July 2020, Santa Maria di Pulsano, Monte Sant’Angelo (Foggia), rocky vegetation, 41.674028° N–15.909410° E, 395 m a.s.l., *obs.* E.V. Perrino; 28 May 2020, Bosco Difesa Grande, Gravina in Puglia (Bari), uncultivated, 40.749207° N–16.375085° E, 381 m a.s.l., *leg. et det.* E.V. Perrino; 29 May 2020, Bosco Scoparella, Ruvo di Puglia (Bari), Garrigue, 41.015204° N–16.424678° E, 370 m a.s.l., *obs.* E.V. Perrino.

Note. *Isatis tinctoria* subsp. *tinctoria* is reported as an invasive alien archaeophyte in Puglia [43]. However, this taxon was never recorded in the relevant literature on semi-natural grassland vegetation in Puglia (e.g., [103,104]) or was recorded as a companion species in single relevés (e.g., [105], a single occurrence in a garrigue in the province of Taranto). In addition, our (R.P.W., E.V.P., and P. Medagli *in verb*.) surveys throughout the Apulian region allow us to state that the status reported in Portal to the Flora of Italy [43] was erroneous, and that actually the species is naturalized alien in Puglia.

**58.**  ***Jaborosa integrifolia* Lam.**

Solanaceae—Neophyte—South America—Rhizomatose geophyte

*Change in status for Sardegna and for Italy: from naturalized alien to invasive alien (invasive alien)*

*Specimina:* 12 July 2023, Via Porto Botte, San Giovanni Suergiu, Sud Sardegna (SU), Meadows along the cycle path, 39.115238° N–08.520295° E, 34 m a.s.l., *leg. et det.* G. Bacchetta, G. Calvia, F. Mascia (Herb. Calvia, CAG); 27 July 2023, Batteria Boggio, Pula (Cagliari), fallow land, meadows and roadsides, 38.999333° N–09.023535° E, 21 m a.s.l., *leg. et det.* G. Bacchetta, G. Calvia, F. Mascia (Herb. Calvia, CAG).

Note. In Sardegna, *J. integrifolia* was recently categorized as a naturalized alien [36]. However, in certain areas of the south-west part of the island, it is increasingly displaying a significant invasive potential. In the town of San Giovanni Suergiu (Sud Sardegna province), this taxon rapidly colonizes abandoned pastures, meadows adjacent to the cycle path, as well as uncultivated lands and roadsides. Moreover, close to Nora (Pula, Cagliari), the taxon is also spreading to neighboring localities from Batteria Boggio, where it was previously reported. A detailed analysis has revealed a more widespread presence.

**59.**  ***Jacaranda mimosifolia* D.Don**

Bignoniaceae—Neophyte—South America—Scapose phanerophyte

*First record for Calabria (casual alien)*

*Specimina:* 11 November 2021, quartiere Sant’Anna, Reggio Calabria, roadside/sidewalk, 38.101913° N–15.643644° E, 24 m a.s.l., *leg. et det.* V.L.A. Laface (REGGIO); 15 June 2022, Feo di Vito, Reggio Calabria, in a wall in the balcony of the AGRARIA Department, 38.120835° N–15.668006° E, 138 m a.s.l., *leg. et det.* V.L.A. Laface (REGGIO); 20 February 2022, Via Argine Sinistro Calopinace, Reggio Calabria, retaining wall of the Calopinace fiumara, 38.101249° N–15.635796° E, 10 m a.s.l., *leg. et det.* C.M. Musarella (REGGIO).

*Observatum:* 11 February 2023, Fiumara Calopinace, Reggio Calabria, river bed, 38.101204° N–15.637219° E, 13 m a.s.l., *obs.* V.L.A. Laface.

Note. *Jacaranda mimosifolia* is reported as a casual alien in Sardegna, Sicilia and Campania [43], there is a herbarium specimen for the region of Puglia at the Herbarium Centrale Italicum (H.C.I.) in Firenze [106]. The many individuals observed grow near mature plants cultivated for street trees.

**60.**  ***Kerria japonica* (L.) DC.**

Rosaceae—Neophyte—China, Japan and Korea—Caespitose phanerophyte

*First record for Calabria (casual alien)*

*Specimen:* 28 August 2022, Località Case Adamo, Melia di San Roberto (Reggio Calabria), roadside, 38.230997° N–15.742205° E, 636 m a.s.l., *leg. et det.* V.L.A. Laface (REGGIO).

Note. *Kerria japonica*, commonly called “Japan rose” [107], is an endemic species of China and Japan; it has been widely cultivated and used as an ornamental plant both in Europe and in the USA for decades, especially in parks and gardens [108]. According to the Portal to the Flora of Italy [43], the species occurs only in the northern regions of Italy, except Liguria and Valle d’Aosta. This is the first record for southern Italy.

**61.**  ***Lampranthus roseus* (Willd.) Schwantes**

Aizoaceae—Neophyte—Cape Province—Succulent chamaephyte

*First record for Calabria (casual alien)*

*Specimen:* 14 May 2021, Ortì Inferiore, Reggio Calabria, roadside, 38.145668° N–15.715387° E, 638 m a.s.l., *leg. et det.* V.L.A. Laface (REGGIO).

Note. *Lampranthus roseus* is cultivated for ornamental purposes [109]. The collected individual was growing in a crack between the wall and the road where it probably originated from seeds coming from plants grown on nearby balconies. In Italy, the species is reported as a casual alien in Campania, Puglia, Sardegna and Toscana [43].

**62.**  **Lantana montevidensis (Spreng.) Briq.**

Verbenaceae—Neophyte—South America—Nanophanerophyte

*First record for Basilicata (casual alien)*

*Change in status for Calabria and for Italy: from casual alien to naturalized alien (naturalized alien)*

*Specimina:* 12 April 2022, SS 18 near a road junction, Tortora, Cosenza, abandoned rest area at the road junction, 39.922464° N–15.770852° E, 21 m a.s.l., *leg. et det.* Fascetti S., Potenza G., Rosati L. (HLUC); 5 June 2022, Cirella, Diamante, Cosenza, vegetation behind the dunes, 39.71792° N–15.809932° E, 6 m a.s.l., *leg. et det.* Fascetti S., Potenza G., Rosati L. (HLUC); 12 July 2023, Via Santa Venere, Fiumicello, Maratea, Potenza, road slope, 39.99511° N–15.703677° E, 55 m a.s.l., *leg. et det.* Fascetti S., Potenza G., Rosati L. (HLUC).

Note. *Lantana montevidensis* is considered highly invasive in the southern United States, Hawaii and Australia [110] because it is rapidly replacing the pasture’s native plants on shallow, dry, stony soils. This species resists prolonged periods of heat and drought and in recent years, it has been observed propagating along the southern Tyrrhenian coast together with *L. camara* on roadsides and in urban green areas of the coast. It was reported as a casual alien in Calabria [52], Sardegna [111] and Liguria regions [112].

**63.**  ***Leucaena leucocephala* (Lam.) de Wit subsp. *glabrata* (Rose) Zárate**

Fabaceae—Neophyte—Central America and Mexico—Scapose phanerophyte

*Change in status for Calabria and for Italy: from naturalized alien to invasive alien (invasive alien)*

*Observata:* 3 October 2022, Lungomare, Reggio Calabria, seedlings and small trees on the beach and pavement cracks, 38.1121531° N–15.6460564° E, 2 m a.s.l., *obs.* A. Capuano; 23 March 2023, Via Graziella, just below the Faculty of Engineering, Reggio Calabria, uncultivated vegetation by the road, 38.1219683° N–15.6648376° E, 59 m a.s.l., *obs.* A. Capuano; 14 July 2023, Pellaro, Reggio Calabria, in the cracks in the sidewalks and flower beds all around the back of the sports hall, 38.025719° N–15.649426° E, 3 m a.s.l., *obs.* C.M. Musarella; 14 July 2023, Pantanello di Pellaro, Reggio Calabria, along the railway’s crushed stone embankment, 38.033020° N–15.656272° E, 9 m a.s.l., *obs.* C.M. Musarella; 14 July 2023, Pantanello di Pellaro, Reggio Calabria, roadside, 38.033964° N–15.656604° E, 7 m a.s.l., *obs.* C.M. Musarella; 14 July 2023, Pantanello di Pellaro, Reggio Calabria, along the railway’s crushed stone embankment, 38.034559° N–15.656758° E, 8 m a.s.l., *obs.* C.M. Musarella; 14 July 2023, Pantanello di Pellaro, Reggio Calabria, along the railway’s crushed stone embankment, 38.040114° N–15.657898° E, 8 m a.s.l., *obs.* C.M. Musarella; 14 July 2023, San Leo di Pellaro, Reggio Calabria, roadside, 38.044224° N–15.655798° E, 11 m a.s.l., *obs.* C.M. Musarella; 14 July 2023, Pellaro, Reggio Calabria, roadside, 38.036398° N–15.659248° E, 15 m a.s.l., *obs.* C.M. Musarella; 14 July 2023, Gallina, Reggio Calabria, roadside, 38.086178° N–15.680670° E, 243 m a.s.l., *obs.* C.M. Musarella; 14 July 2023, Gallina, Reggio Calabria, roadside, 38.086037° N–15.680701° E, 243 m a.s.l., *obs.* C.M. Musarella; 12 August 2023, Pellaro, Reggio Calabria, roadside, 38.032297° N–15.657437° E, 14 m a.s.l., *obs.* C.M. Musarella; 12 August 2023, Ravagnese, Reggio Calabria, on the asphalt in the central reservation of State Road 106 (SS 106), 38.062000° N–15.662231° E, 41 m a.s.l., *obs.* C.M. Musarella; 12 August 2023, Pellaro, Reggio Calabria, roadside, 38.033117° N–15.657929° E, 19 m a.s.l., *obs.* C.M. Musarella; 12 August 2023, Pellaro, Reggio Calabria, roadside, 38.033117° N–15.657929° E, 19 m a.s.l., *obs.* C.M. Musarella; 12 August 2023, Pellaro, Reggio Calabria, roadside, 38.036442° N–15.659249° E, 15 m a.s.l., *obs.* C.M. Musarella; 12 August 2023, Pellaro, Reggio Calabria, roadside, 38.037198° N–15.659309° E, 20 m a.s.l., *obs.* C.M. Musarella; 12 August 2023, Ravagnese, Reggio Calabria, roadside along the highway connection to the airport, 38.073388° N– 15.655525° E, 20 m a.s.l., *obs.* C.M. Musarella; 13 August 2023, Quartiere Lume, Pellaro, Reggio Calabria, roadside, 38.018663° N–15.651368° E, 29 m a.s.l., *obs.* C.M. Musarella; 11 November 2023, along the State Road 106 (SS 106) at the bridge over Ponzo creek, Santa Caterina dello Ionio (Catanzaro), riparian vegetation along the water course, 38.5605390° N–16.5699725° E, 9 m a.s.l., *obs.* A. Capuano, G. Montepaone, G. Caruso; 13 November 2023, roundabout close to the Lamezia Terme airport, Bellafemmina (Catanzaro), scrubland along the road, 38.9117072° N–16.2564866° E, 14 m a.s.l., *obs.* A. Capuano.

Note. The native range of *Leucaena leucocephala* subsp. *glabrata* is from Mexico to Honduras [61], reported for Italy only in some regions as a casual or naturalized alien [43]. This plant was first reported as a casual alien for the Calabria region (only in the Metropolitan City of Reggio Calabria) and continental Italy by Musarella et al. [59]. It was later reported as a naturalized alien for the region by Stinca et al. [58], because many adults at different growth stages, with different inflorescences and mature fruits, were found in new and old localities also in other Calabrian provinces and all producing many seedlings. This taxon continues to spread rapidly along city roadsides, highways and railways, as well as in the cracks of the sidewalks, germinating easily from the many scattered seeds. The finding of new adult individuals, apparently having an age of more than 10 years, and juveniles widespread near and far from them at already known sites and in new localities in the provinces of Reggio Calabria and Catanzaro justifies properly considering *L. leucocephala* subsp. *glabrata* as an invasive alien for the region and, then, for Italy.

**64.**  **Lobelia erinus L.** [≡*Dortmanna erinus* (L.) Kuntze, ≡*Rapuntium erinus* (L.) Mill.]

Campanulaceae—Neophyte—Central and South Africa—Scapose therophyte

*First record for Calabria (casual alien)*

*First record for Molise (casual alien)*

*Specimen:* 23 June 2023, Largo San Domenico, Isernia, roadside/sidewalk, 41.593600° N–14.229586° E, 455 m a.s.l., *leg. et det.* E. Bajona (FI).

*Observata:* 1 May 2020, Ortì Superiore, Reggio Calabria, abandoned field and roadside, 38.150949° N–15.730260° E, 665 m a.s.l., *obs*. V.L.A. Laface; 15 May 2020, Pellaro, Reggio Calabria, in a crack of a wall, 38.021617° N–15.647807° E, 14 m a.s.l., *obs.* C.M. Musarella.

Note. Species introduced in Europe as an ornamental plant with several varieties, *Lobelia erinus* easily spreads in urban environments where it is found as a casual alien species [43]. The specimen observed in Pellaro has grown in a crack in a wall during its flowering period. Both *observata* individuals above reported probably come from the neighboring house, where this species is commonly cultivated.

**65.**  ***Lycium barbarum* L.**

Solanaceae—Neophyte—China—Nanophanerophyte

*First record for Calabria (casual alien)*

*Specimen:* 20 January 2022, contrada Capperi, Badolato (Catanzaro), synanthropic recovery vegetation of highly altered and disturbed environments, 38.560739° N–16.571149° E, 10 m a.s.l., *leg. et det.* G. Mei (Herbarium Mei).

Note. A flowering individual of *L. barbarum* was found in the center of the *fiumara* not far from a horticultural area where a well-developed plant with flowers and fruit was observed.

**66.**  ***Melia azedarach* L.**

Meliaceae—Neophyte—South Asia, Malaysia, Indonesia and Australia—Scapose phanerophyte

*Change in status for Calabria and for Italy: from naturalized alien to invasive alien (invasive alien)*

*Specimina:* 28 July 2021, Copanello di Stalettì by the mouth of Alessi river (Catanzaro), amongst windbreak hedges along the road, 38.7692208° N–16.5673852° E, 3 m a.s.l., *leg.* A. Capuano, *det.* A. Capuano, G. Caruso (REGGIO); 8 August 2022, Gagliano old town along Via Filippo Smaldone, Catanzaro, roadside and sidewalk crubs along with *Lantana camara s.l.*, 38.920968° N–16.561711° E, 377 m a.s.l., *leg.* A. Capuano, *det.* A. Capuano, G. Caruso (REGGIO); 12 August 2022, Via Biagio Miraglia, Catanzaro, roadside at the base of a wall, 38.910854° N–16.569096° E, 313 m a.s.l., *leg.* A. Capuano, *det.* A. Capuano, G. Caruso (REGGIO); 18 October 2023, quartiere Mater Domini, Catanzaro, roadside at the base of a retaining wall, 38.914335° N–16.567521° E, 352 m a.s.l., *leg.* A. Capuano, *det.* A. Capuano, G. Caruso (REGGIO); 6 December 2023, Squillace Lido along the State Road 106 (SS 106) (Catanzaro), roadside amongst herbaceous vegetation, 38.782136° N–16.570572° E, 9 m a.s.l., *leg. et det.* A. Capuano, G. Caruso (REGGIO).

*Observata:* 17 September 2022, Reggio Calabria, on the asphalt in the central reservation of State Road 106 (SS 106), 38.096185° N–15.659083° E, 89 m a.s.l., *obs.* C.M. Musarella; 17 September 2022, Reggio Calabria, on the asphalt in the central reservation of State Road 106 (SS 106), 38.109437° N–15.660942° E, 93 m a.s.l., *obs.* C.M. Musarella; 17 September 2022, Reggio Calabria, in several cracks of the walksides, 38.116145° N– 15.658228° E, 51 m a.s.l., *obs.* C.M. Musarella; 25 June 2022, Fiumara di Melito, Melito di Porto Salvo (Reggio Calabria), at the edge of the *fiumara*, 37.925015° N–15.759500° E, 27 m a.s.l., *obs.* V.L.A. Laface; 8 September 2022, Straci, Condofuri Marina, Condofuri (Reggio Calabria), roadside, 37.922739° N–15.840596° E, 13 m a.s.l., *obs.* V.L.A. Laface; 15 October 2022, Pellaro, along the State Road 106 (SS 106), Reggio Calabria, roadside, 38.034957° N–15.658482° E, 15 m a.s.l., *obs.* V.L.A. Laface; 12 January 2023, Via Demetrio Tripepi, Reggio Calabria, roadside and sidewalk, 38.114564° N–15.652050° E, 32 m a.s.l., *obs.* V.L.A. Laface; 23 February 2023, Gallico Superiore, Reggio Calabria, citrus grove abandoned for several years, 38.171052° N–15.672567° E, 108 m a.s.l., *obs.* V.L.A. Laface; 23 February 2023, ponte fra Sambatello e San Giovanni di Sambatello, Reggio Calabria, at the edge of an abandoned citrus grove, 38.175321° N–15.685524° E, 182 m a.s.l., *obs.* V.L.A. Laface; 6 May 2023, Autostrada del Mediterraneo (A2), Reggio Calabria, roadside, 38.109333° N–15.660776° E, 93 m a.s.l., *obs.* V.L.A. Laface; 13 August 2023, Quartiere Lume, Pellaro, Reggio Calabria, coming out of a rainwater drainage grate, 38.018702° N–15.651399° E, 28 m a.s.l., *obs.* C.M. Musarella; 22 August 2023, Archi Carmine, Reggio Calabria, roadside, 38.156424° N–15.665198° E, 50 m a.s.l., *obs.* V.L.A. Laface.

Note. *Melia azedarach,* introduced as an ornamental species, is commonly cultivated as a street tree throughout the region. It was first reported in Calabria (Cosenza Province) as a casual alien species in 2015 [113] and subsequently considered a naturalized alien in 2021 [58]. In recent years, it has been noticed that spontaneous seedlings observed in previous years have quickly and successfully colonized numerous suburban and urban areas, spreading mainly in disturbed roadside habitats and urban open spaces, but have also been observed along some *fiumarerivers* that have been heavily disturbed by human activities and roadsides. This species is very well adapted to the Mediterranean climate [114]; in fact, in South Africa, it is considered a highly invasive alien species with extremely rapid growth, listed as one of the top ten invasive alien plants in terms of the area in which it grows [115]. *Melia azedarach* is considered, moreover, one of the most important exotic plants transforming natural habitats [116]. The finding of new adult individuals, apparently having an age of more than 10 years, and juveniles widespread near and far from them at already known sites and in new localities in the metropolitan city of Reggio Calabria, justifies properly considering *M. azedarach* as an invasive alien for the region and, then, for Italy.

**67.**  ***Moeroris tenella* (Roxb.) R.W.Bouman** [=*Phyllanthus tenellus* Roxb.]

Phyllanthaceae—Neophyte—East Africa and Madagascar—Scapose therophyte

*First record for Sardegna (casual)*

*Specimen:* 30 August 2022, Valle di Palabanda, Cagliari, wet and shady synanthropic habitats, 39.221480° N–09.110308° E, 43 m a.s.l., *leg. et det.* L. Podda, G. Bacchetta, G. Calvia (CAG).

Note. Many individuals of *Moeroris tenella* were discovered as weeds in various ornamental plant pots within the Hortus Botanicus Karalitanus (University of Cagliari). Subsequently, scattered plants were observed in neighboring sites of the Palabanda Valley (Cagliari) in open fields, particularly in damp and shaded environments. *Moeroris tenella* was previously documented in only three other regions of Italy: Liguria and Calabria as casual occurrences, and in Sicilia as naturalized alien, as reported by the Portal to the Flora of Italy [43].

**68.**  ***Momordica charantia* L.**

Cucurbitaceae—Neophyte—Paleotropical—Scandense therophyte

*First record for Sardegna (casual alien)*

*Specimen:* 28 May 2022, Tanca Manna, Capoterra (Cagliari), agricultural and rural habitats, 39.173267° N–09.001946° E, 6 m a.s.l., *leg. et det.* F. Mascia, L. Podda, G. Bacchetta (CAG).

Note. *Momordica charantia* is an Asian plant cultivated for food purposes and rarely escaped cultivation in Emilia Romagna and Lazio, as previously observed by Alessandrini and Montanari [117] and Lucchese [118]. A few individuals that escaped cultivation were found along roadsides and hedges in the colluvial area of Tanca Manna, in Capoterra (Cagliari).

**69.**  ***Myoporum laetum* G. Forst.** [≡*Myoporum pubescens* G. Forst.]

Scrophulariaceae—Neophyte—New Zealand—Caespitose phanerophyte

*First record for Sicilia (casual alien)*

*Specimen:* 12 April 2023, Contrada Oliastrello, Ustica (Palermo), base of a wall, 38.711301° N–13.190106° E, 70 m a.s.l., *leg.* G. Domina, *det.* G. Domina, E. Di Gristina, G. Barone (SAF100127).

Note. *Myoporum laetum* is used for ornamental purposes. One individual was found at the base of a wall, probably coming from nearby adult fruiting plants. The species is present in Italy as a naturalized alien in Sardegna, while in the past, it was reported by mistake for Puglia and Toscana [43].

**70.**  ***Nassella neesiana* (Trin. & Rupr.) Barkworth** [≡*Stipa neesiana* Trin. & Rupr.;—*Stipa mucronata* auct. Fl. Ital., non Kunth;—*Nassella mucronata* auct. Fl. Ital., non (Kunth) R.W.Pohl;—*Stipa setigera* auct. Fl. Ital., non J.Presl]

Poaceae—Neophyte—South America—Caespitose hemicryptophyte

*Change in status for Liguria: from casual alien to naturalized alien (naturalized alien)*

*Specimen:* 27 June 2023, Lerca, Via al Golf, Cogoleto (Genova), barren meadow, 44.405316° N–08.642843° E, 129 m a.s.l., SE, *leg*. G. Galasso, *det*. E. Banfi, G. Galasso (MSNM barcodes MSNM52533, MSNM52534, MSNM52535).

Note. *Nassella neesiana* was observed abundant also in Chiappino (Cogoleto). The species is known to be alien in the USA (Alabama), Europe (Great Britain, Germany, Italy, Iberian and Balkan Peninsula), South Africa, east Australia, Tasmania, New Zealand [61]. In Italy, it is currently a naturalized alien in Lazio and Toscana and a casual alien in Calabria, Emilia-Romagna, Liguria and Veneto (Portal to the Flora of Italy, 2023). Now, it must be considered definitely a naturalized alien in Liguria as well, where it has greatly spread compared to the previous indication by Verloove et al. [119].

**71.**  ***Nerium oleander* L. subsp. *oleander* cultivar ‘Pink Beauty’**

Apocynaceae—Neophyte—Mediterranean basin and South Asia (Culton)—Caespitose phanerophytes

*First record for Puglia (casual alien)*

*Observata:* 30 May 2020, Torre d’Orta, Monopoli, Bari, shrub vegetation, 40.967844° N–17.265433° E, 22 m a.s.l., *obs.* E.V. Perrino; 30 May 2020, East of Torre Incina, Monopoli, Bari, uncultivated, 40.976742° N–17.260776E, 7 m a.s.l., *obs.* E.V. Perrino.

Note. In Puglia, it is an alien taxon because it does not grow in its typical environment as in other regions of south Italy, such as Calabria, where, with some species of the genus *Tamarix*, it forms a protected habitat along the river. In Puglia this taxon has escaped cultivation as an ornamental cultivar: in fact, it is observed in different environments from its ecology. The *observation* above refers to the “Pink Beauty” cultivar, which is one of the most widespread varieties on the market and widely used in Puglia, not only in public gardens but also along the highways. It has a simple pink flower with a diameter of about 6 cm. It was observed in two coastal places in the municipalities of Monopoli (Bari) in two different vegetations: within the shrub plant community with high coverage of *Pistacia lentiscus* at Torre d’Orta, and near Torre Incina in uncultivated vegetation with sub-nitrophilous species.

**72.**  ***Ocimum basilicum* L.**

Lamiaceae—Archaeophyte—South East Asia, Malaisia, Indonesia and Australia—Scapose therophyte

*First record for Calabria (casual alien)*

*Specimina:* 6 August 2022, old town, Verbicaro (Cosenza), roadside/sidewalk, 39.755030° N–15.911812° E, 421 m a.s.l., *leg. et det.* Fascetti S., Potenza G., Rosati L. (HLUC); 27 September 2023, Via Salita Melissari, Reggio Calabria, roadside, 38.120114° N–15.660167° E, 52 m a.s.l., *leg. et det.* V.L.A. Laface, G. Mazzacuva (REGGIO); 20 September 2023, Via Provinciale, Mosorrofa, Reggio Calabria, roadside, 38.097063° N–15.718405° E, 397 m a.s.l., *leg. et det.* V.L.A. Laface, G. Mazzacuva (REGGIO).

Note. *Ocimum basilicum* is universally cultivated as an annual herbaceous plant originating from the Asian continent and it is used as a culinary herb [120]. The species has been reported in the alien flora of many Italian regions as a casual alien [43]. It is a synanthropic species occasionally found in various types of environments such as rural and ruderal [118,121,122] and riverbeds [32,123]. The individuals observed grew in a ruderal environment and originated from plants grown nearby.

**73.**  ***Oenothera gaura* W.L.Wagner & Hoch**

Onagraceae—Neophyte—North America—Biennial hemicryptophyte

*First record for Sardegna (casual alien)*

*Specimen:* 3 August 2023, Margine Rosso, Quartu S. Elena (Cagliari), coastal habitats, 39.228301° N–09.228600° E, 0 m a.s.l., *leg. et det.* G. Bacchetta, G. Calvia, L. Podda (CAG).

Note. Several individuals of *O. gaura* were found in the locality of Margine Rosso in Quartu S. Elena town, along a roadside and on sandy places, and probably escaped from the gardens.

**74.**  ***Oenothera stucchii* Soldano**

Onagraceae—Neophyte—Artificial hybrid (Culton)—Biennial hemicryptophyte

*First record for Puglia (naturalized alien)*

*Specimina:* 14 August 2019, Isthmus of Varano, Ischitella (Foggia), sandy environment in the backdune, 41.919483° N–15.792990° E, 0 m a.s.l., *leg. et det.* D. Bonsanto, N. Biscotti (FI, Herb. Biscotti, Herb. Bonsanto); 14 August 2019, in località Capojale, Cagnano Varano (Foggia), sandy soil, 41.911308° N–15.683642° E, 0 m a.s.l., *leg. et det.* D. Bonsanto, N. Biscotti (FI, Herb. Biscotti, Herb. Bonsanto).

Note. In Italy, *O. stucchii* is reported as a naturalized or invasive alien for many regions, but not in Puglia [43]. Very dense populations are found in the sandy shoreline of Isola Varano, and in the backdune near a crop in the Capojale area. Inside the stands, the species is fastly spreading.

**75.**  ***Paspalum exaltatum* J.Presl**

Poaceae—Neophyte—South America—Caespitose hemicryptophyte

*Change in status for Liguria and for Italy: from casual alien to naturalized alien (naturalized alien)*

*Specimen:* 27 June 2023, Via Ronco, Chiappino, Cogoleto (Genova), roadside, 44.394140° N–08.640292° E, 111 m a.s.l., E, *leg.* G. Galasso, *det.* E. Banfi, G. Galasso (MSNM barcodes MSNM52545, MSNM52546, MSNM52547, MSNM52548).

Note. Currently, *P. exaltatum* is present extra patriam only in Italy (Liguria), where in recent decades it has demonstrated an ability to robustly expand on the edges of the road in question starting from the report by Verloove & Reynders [124] and presumably elsewhere in the same territory.

**76.**  ***Perilla frutescens* (L.) Britton** [≡*Ocimum frutescens* L.]

Lamiaceae—Neophyte—East and South Asia—Scapose therophyte

*First record for Calabria (casual alien)*

*Specimina:* 1 October 2021, Viale della Libertà, Motta San Giovanni (Reggio Calabria), roadside, 38.000528° N–15.689403° E, 416 m a.s.l., *leg. et det.* V.L.A. Laface (REGGIO); 19 September 2022, Via dei Tre Mulini, Reggio Calabria, roadside and cracks in the wall, 38.120766° N–15.660292° E, 59 m a.s.l., *leg. et det.* V.L.A. Laface (REGGIO); 9 July 2022, Patarriti, Motta San Giovanni (Reggio Calabria), roadside, 38.031496° N–15.695109° E, 413 m a.s.l., *leg. et det.* V.L.A. Laface (REGGIO); 26 September 2022, via dei Tre Mulini, Reggio Calabria, by the roadside at the base of a wall, 38.120790° N–15.660222° E, 55 m a.s.l., *leg.* A. Capuano, *det.* A. Capuano, G. Caruso (REGGIO, Herbarium Capuano).

Note. The individuals of *P. frutescens* observed in the cracks of a wall and in drainage channels probably come from plants grown on the nearby balconies and gardens. *Perilla frutescens* is widely cultivated, especially in its native area for medical, culinary, and ornamental purposes [125,126]. Based on the morphology and color of the leaves, the material collected can be referred to as the var. *crispa* (Bentham) Deane ex Bailey [125].

**77.**  ***Persicaria capitata* (Buch.-Ham. ex D.Don.) H.Gross**

Polygonaceae—Neophyte—South Asia—Reptant hemicryptophyte

*First record for Basilicata (casual alien)*

*Change in status for Calabria from casual alien to naturalized alien (naturalized alien)*

*Specimina:* 2 June 2023, old town, Località S. Biagio, Maratea (Potenza), cracks in the walls of ancient abandoned houses, 39.993142° N–15.720169° E, 312 m a.s.l., *leg. et det.* Fascetti S., Potenza G., Rosati L. (HLUC); 30 March 2023, old town of Scalea (Cosenza), cracks in stone walls, 39.81591° N–15.791199° E, 24 m a.s.l., *leg. et det.* Fascetti S., Potenza G., Rosati L. (HLUC); 20 July 2023, Carcere dell’Impresa, S. Maria del Cedro (Cosenza), stone paving in a garden, 39.745625° N–15.821596° E, 30 m a.s.l., *leg. det.* Fascetti S., Potenza G., Rosati L. (HLUC).

Note. *Persicaria capitata* was reported in many peninsular regions, in Sicilia and in Sardegna [43]. In particular, this species was known as a naturalized alien in Campania [127] and as a casual alien in Puglia [128] and in Calabria [52]. Therefore, with this new finding, the last three regions stand in territorial continuity with Basilicata, where *P. capitata* occurs in urban and ruderal environments on rocky or debris substrates, as in neighboring regions.

**78.**  ***Persicaria senegalensis* (Meisn.) Soják**

Polygonaceae—Neophyte—Africa, Madagascar and Arabian Peninsula—Scapose therophyte

*First record for Continental Europe (naturalized alien)*

*Specimina:* 5 September 2023, Saline Joniche, Montebello Jonico (Reggio Calabria), drainage for water at the roadside, 37.940408° N–15.708912° E, 12 m a.s.l., *leg.* V.L.A. Laface, G. Mazzacuva, *det.* V.L.A. Laface, G. Mazzacuva, C.M. Musarella (REGGIO); 27 October 2023, Saline Joniche, Montebello Jonico (Reggio Calabria), beach, 37.939389° N–15.707500° E, 1 m a.s.l., *leg.* G. Mazzacuva, *det.* V.L.A. Laface, G. Mazzacuva (REGGIO).

Note. The numerous individuals of *P. senegalensis* observed grow inside a water drainage channel, where the flow is constant: the population also occupies a big part of the beach below. The species was reported for the first time in Europe in Crete Island [129] and in Italy only in the Pantelleria Island [130]: therefore, this is the first report for continental Europe. *Persicaria senegalensis* is a species that attracts particular attention because it has a very high reproductive capacity, both gamic and agamic, which allows it to rapidly colonize very large areas near water.

**79.**  ***Petroselinum crispum* (Mill.) Fuss**

Apiaceae—Archaeophyte—Algeria, Greece, Morocco and Yugoslavia—Biennial hemicryptophyte

*Change in status for Puglia: from casual alien to naturalized alien (naturalized alien)*

*Specimen:* 10 August 2023, Chianche Lisce, Vico del Gargano (Foggia), incolto, 41.904593° N–15.940482° E, 461 m a.s.l., *leg. et det.* D. Bonsanto, N. Biscotti (FI, Herb. Bonsanto).

Note. The specimen reported of *P. crispum* comes from dense and widespread populations in an uncultivated olive grove. This species is reported for all Italian regions except Valle d’Aosta. It is listed as a casual alien for Puglia, as for most other regions, except Sardegna and Trentino Alto Adige, where it is considered a naturalized alien [43]: however, considering our observations, in the reported site, it is a naturalized alien.

**80.**  ***Phoenix canariensis* H.Wildpret**

Arecaceae—Neophyte—Canary Is.—Scapose phanerophyte

*Change in status for Calabria: from casual alien to naturalized alien (naturalized alien)*

*Specimina:* 2 July 2021, Copanello di Stalettì by the mouth of Alessi river (Catanzaro), amongst windbreak hedges along the road, 38.7692208° N–16.5673852° E, 3 m a.s.l., *leg.* A. Capuano, *det.* A. Capuano, G. Caruso (REGGIO); 2 July 2021, Villaggio Cala Verde, Copanello Lido, Stalettì (Catanzaro), amongst hedges, 38.7682246° N–16.5662948° E, 2 m a.s.l., *leg.* A. Capuano, *det.* A. Capuano, G. Caruso (REGGIO); 2 September 2022, Via Alfonso Frangipane, Catanzaro, on a wall along with *Cupressus sempervirens* L., 38.9126182° N–16.5713592° E, 338 m a.s.l., *leg.* A. Capuano, *det.* A. Capuano, G. Caruso (REGGIO); 9 October 2022, green area in front of Mater Domini church, Catanzaro, amongst hedges, 38.916498° N–16.568390° E, 374 m a.s.l., *leg.* A. Capuano, *det.* A. Capuano, G. Caruso (REGGIO); 7 April 2023, Cirella cliff, Diamante (Cosenza), pockets of debris on rocks, 39.927421° N–15.76467° E, 7 m a.s.l., *leg. et det.* Fascetti S., Potenza G. (HLUC); 30 September 2023, Via Antonio Lombardi, Catanzaro, slope with *Arundo plinii* Turra, 38.902475° N–16.580901° E, 294 m a.s.l., *leg.* A. Capuano, *det.* A. Capuano, G. Caruso (REGGIO); 30 September 2023, Via Luigi Pascali, Catanzaro, roadside, 38.9147383° N–16.5898245° E, 358 m a.s.l., *leg.* A. Capuano, *det.* A. Capuano, G. Caruso (REGGIO); 5 October 2023, Viale Tommaso Campanella, Catanzaro, under some trees and bushes by the road, 38.909163° N–16.573642° E, 314 m a.s.l., *leg.* A. Capuano, *det.* A. Capuano, G. Caruso (REGGIO); 7 October 2023, Viale Isonzo, Catanzaro, uncultivated field by the road, 38.8604340° N–16.6050880° E, 63 m a.s.l., *leg.* R. Ritrovato, A. Capuano, *det.* A. Capuano, G. Caruso (REGGIO); 7 October 2023, along viale Isonzo by the Fiumarella river embankment, Catanzaro, 38.841286° N–16.610401° E, 31 m a.s.l., *leg.* R. Ritrovato, A. Capuano, *det.* A. Capuano, G. Caruso (REGGIO).

*Observata:* 28 June 2018, Vasche di Cassiodoro, ZSC Scogliera di Stalettì, Stalettì (Catanzaro), in the crevices of the rocks, 38.761117° N–16.571154° E, 4 m a.s.l., E, *obs.* C.M. Musarella, G. Spampinato; 28 July 2021, Copanello Lido, Stalettì (Catanzaro), undergrowth of *Pinus pinea* L. plantation, 38.767783° N–16.562679° E, 12 m a.s.l., *obs.* A. Capuano; 30 December 2021, Caminia, Stalettì (Catanzaro), drainage channel along with *Parthenocissus tricuspidata* (Siebold & Zucc.) Planch., 38.7459877° N–16.5559636° E, 64 m a.s.l., *obs.* A. Capuano; 30 September 2022, between AGRARIA and Engineering University Departments, Reggio Calabria, drainage channel in a mixed alien-native plant community, 38.119728° N–15.666827° E, 87 m a.s.l., *obs.* A. Capuano; 10 October 2022, Brancaleone Marina, Brancaleone (Reggio Calabria), abandoned field, 37.951786° N–16.089709° E, 9 m a.s.l., *obs*. V.L.A. Laface; 23 October 2022, Fiumara Calopinace, argine dx, Reggio Calabria, at the edge of the Fiumara bed, 38.101682° N–15.644444° E, 29 m a.s.l., *obs*. V.L.A. Laface; 29 November 2022, entrance road of Lamezia Terme Airport (Catanzaro), roadside at the base of *Pinus pinea* L. trees, 38.9091132° N–16.2543556° E, 10 m, *obs.* A. Capuano, G. Caruso; 29 November 2022, entrance road of Lamezia Terme Airport (Catanzaro), roadside at the base of *Juglans regia* L. tree and amongst *Rubus ulmifolius* Schott shrubland in the nearby field, 38.9108580° N–16.2519688° E, 10 m a.s.l., *obs.* A. Capuano, G. Caruso; 29 November 2022, along the State Road 18 (SS 18), Lamezia Terme (Catanzaro), under some trees by the roadside 38.9108124° N–16.2603866° E, 20 m a.s.l., *obs.* A. Capuano, G. Caruso; 12 January 2023, escarpment between via Piave and the Ferrovie della Calabria station, Catanzaro, *Robinia pseudoacacia* scrubland, 38.9135041° N–16.5845657° E, 350 m a.s.l., *obs.* A. Capuano, G. Caruso; 10 March 2023, Praia a Mare (Cosenza), in the crevices of *Platanus* trees, 39.896568° N–15.780784° E, 7 m a.s.l., E, *obs.* V.L.A. Laface, C.M. Musarella, G. Spampinato; 17 September 2023, Roccelletta di Borgia by the mouth of Corace river (Catanzaro), on the edge of *Eucalyptus* plantation, 38.812594° N–16.602852° E, 2 m a.s.l., *obs.* A. Capuano, G. Caruso; 17 September 2023, Montepaone Lido by the mouth of Beltrame river (Catanzaro), amongst psammophilous communities, 38.707480° N–16.534393° E, 2 m a.s.l., *obs.* A. Capuano, G. Caruso; 30 September 2023, by the roundabout along Via Gioacchino da Fiore, Catanzaro, slope along with *Vitis rupestris* Scheele, 38.905082° N–16.577862° E, 300 m a.s.l., *obs.* A. Capuano, G. Caruso.

Note. *Phoenix canariensis* was reported as a naturalized alien for the Calabria region by Galasso et al. (2018a), but recently, it changed status from naturalized alien to casual alien [59]. In the Diamante site, the species is present with seven young specimens of different ages, coming from zoochorous dissemination of the seeds of the palm trees grown in the nearby gardens. In addition, new observations were made in different parts of the Calabria region. For this reason, we now consider that *P. canariensis* could be considered a naturalized alien.

*Phoenix canariensis*, native to the Canary Islands, has become a popular ornamental plant in Europe, in the Mediterranean area and also in Australia, California and Florida, where it is largely cultivated as a garden or landscape species in public and private spaces [131,132]. It is a dioecious plant that can produce up to 30.000 dates per year, resulting in a great source of fresh food for the wild avifauna especially in urban environments, both in its native area and outside of its natural range [133,134]. Its abundant fruiting, often conveyed by zoochory, led this palm to spread extensively outside of cultivation, bringing it to develop in a wide range of habitats, from man-made to natural environments often far away from the mother plants. *Phoenix canariensis* was reported as a naturalized alien for the Calabria region by Galasso et al. [27], but recently it changed status from a naturalized alien to a casual alien [59]. In recent years, new observations have been made in different parts of the Calabria region, especially in the Catanzaro province. For this reason, we now consider that *P. canariensis* could be treated as a naturalized alien. In the surveyed stands, *P. canariensis* showed great adaptability in terms of habitats and substrates: we observed populations developing on different soil typologies, from sandy or rocky spots on the coastline to clayey slopes or alluvial sites in the immediate inland. Although several stands host young and non-fruiting plants (at least for the moment), we also found mature specimens actively fruiting and disseminating in the surroundings. Also, some new seedlings were developing very far away from the putatively naturalized alien mother plants. It can be stated that *P. canariensis* is well established in Calabria and further investigations across different areas of the region will reveal new stands. Moreover, the increasing global temperatures will help subtropical floristic elements to find better conditions for their development. Although in recent years, several cultivated plants have been decimated by the Asian palm weevil *Rhynchophorus ferrugineus* (Dryophthoridae), it is expected that the spread of new seedlings and the stabilization of new populations could affect many more native communities in the future, especially in natural environments as already reported for other Italian regions [135,136].

**81.**  ***Phyllostachys aurea* Carrière ex Rivière & C.Rivière**

Poaceae—Neophyte—China and Vietnam—Caespitose phanerophyte

*First record for Basilicata (casual alien)*

*First record for Trentino-Alto Adige (casual alien)*

*Change in status for Puglia: from casual alien to naturalized alien (naturalized alien)*

*Specimina:* 19 August 2023, Rotonda (Potenza), roadside and road, 39.927305° N–16.046336° E, 722 m a.s.l., *leg.* V.L.A. Laface, A. Mammoliti, *det.* V.L.A. Laface (REGGIO); 11 March 2020, San Nicola, Vico del Gargano (Foggia), torrent margin, 41.912349° N–15.950736° E, 160 m a.s.l., *leg.* N. Biscotti, D. Bonsanto, *det.* N. Biscotti, D. Bonsanto (FI); 27 August 2020, Valle del Melaino, Vico del Gargano (Foggia), torrent margin, 41.880536° N–15.950339° E, 30 m a.s.l., *leg.* D. Bonsanto, N. Biscotti, *det.* D. Bonsanto, N. Biscotti (Herb. D. Bonsanto); 26 July 2023, Strada Statale 693 dei Laghi di Lesina e Varano, Ischitella (Foggia) stream bank, 41.905430° N–15.875414° E, 46 m a.s.l., *leg.* D. Bonsanto, N. Biscotti, *det.* D. Bonsanto, N. Biscotti (Herb. D. Bonsanto).

*Observatum:* 23 July 2023, industrial area, Lavis (Trento), roadside, 46.146332° N–11.089351° E, 72 m a.s.l., *obs.* R. Motti.

Note. *Phyllostachys aurea* is recorded as a casual or naturalized alien in many Italian regions, while it shows a distribution gap only in Trentino Alto Adige, Marche, Basilicata and Sardegna [43]. In Basilicata, the collected specimen of this species, cultivated for ornamental purposes, probably comes from a small population, which acts as a hedge in a nearby villa. A small population was also observed in an uncultivated area between the motorway and an industrial area in Trentino Alto Adige and this finding is the first for the region.

**82.**  ***Physalis peruviana* L.**

Solanaceae—Neophyte—Bolivia and Brazil—Scapose hemicryptophyte

*First record for Basilicata (casual)*

*Specimen:* 2 July 2023, Fosso del Gallitello, Potenza (Potenza), riverbank, 40.637734° N–15.783321° E, 696 m a.s.l., *leg. et det.* Fascetti S., Potenza G., Rosati L. (HLUC).

Note. *Physalis peruviana* is an herbaceous perennial species that has been very widely introduced across the world from South America as a cultivated plant for its fruit, as a medicinal plant, and as an ornament. It is reported as a casual alien in several regions and as naturalized in Sicilia [43]. In south Italy, it is recorded as a casual alien previously from the Puglia, Sicilia and Calabria regions [27,111,137]. It is classified as an invasive alien plant at the global level [138]. The species grows wild on sandy soils in fallow fields [28,111], river banks and lake shores [28]. The plants regularly develop flowers and fruits.

**83.**  ***Pinus elliottii* Engelm.**

Pinaceae—Neophyte—South East U.S.A.—Scapose phanerophyte

*First record for Europe (casual alien)*

*Specimen:* 15 October 2022, Scala mala, Putifigari (Sassari), Forestry habitats, 40.587875° N–08.423305° E, 121 m a.s.l., *leg. et det.* F. Mascia, G. Calvia, G. Bacchetta (CAG).

Note. In the past, *Pinus elliottii* has been used for reforestation and public greening in some places on Sardegna island. Renewal phenomena are sporadically observed with plants of all age classes. In the mentioned site, several plants have been observed on roadsides, originating from some nearby roadside tree plantations. According to Euro+Med plantbase [80], the species is not recorded in Europe even as a casual alien, while it is considered a naturalized alien in some parts of Argentina, Australia, Hawaii, New Caledonia, South Africa, and Zimbabwe [139,140,141,142].

**84.**  ***Polanisia dodecandra* (L.) DC. subsp. *trachysperma* (Torr. & A.Gray) Iltis**

Cleomaceae—Neophyte—North America—Scapose therophyte

*Change in status for Toscana and for Italy: from naturalized alien to invasive alien (invasive alien)*

*First record for Umbria (naturalized alien)*

*Specimina:* 4 August 2022, Strada comunale di Lancaia, Pomarance (Pisa), gravel beds, 43.312550° N–10.910470° E, 110 m a.s.l., *leg. et det.* T. Fiaschi, C. Angiolini (SIENA); 30 August 2022, under Ponte Sandro Pertini, Orvieto (Terni), gravel beds, 42.729798° N–12.126354° E, 110 m a.s.l., *leg. et det.* T. Fiaschi, C. Angiolini (SIENA).

Note. *Polanisia dodecandra* subsp. *trachysperma* abundantly colonizes a stretch of the Tiber river, where it invades the EU habitat 3250, threatening the endemic *Santolina etrusca* (Lacaita) Marchi & D’Amato as previously highlighted in nearby areas [143,144]. This taxon is reported for only a few Italian regions: it is considered a naturalized alien in Toscana, Lombardia, Piemonte and Emilia Romagna, and a casual alien in Lazio and Liguria [43]. Very abundant populations (thousands of individuals) were found on the bed of the Possera stream, near the confluence with the Cecina river. Its presence in the area was already known, as reported by Selvi and Bettini [145]. The species is also present on the Trasubbie stream as reported by Frignani et al. [146] and on the Merse river as reported by Landi et al. [147].

**85.**  ***Portulacaria afra* Jacq.**

Didiereaceae—Neophyte—Kenya, Mozambique and South Africa—Succulent phanerophyte

*First record for peninsular Italy (casual alien)*

*Specimen:* 14 August 2023, Reggio Calabria, inside the water drainage channel along the A2 motorway junction, towards Salerno, 38.125144N-15.653063E, 13 m a.s.l., *leg.* C.M. Musarella, *det*. V.L.A. Laface, C.M. Musarella, G. Spampinato (REGGIO).

Note. Several individuals of *Portulacaria afra* were found in a water drainage channel, among other several alien species. They were probably formed from leaves and broken twigs of plants grown on the balconies and terraces of the many apartments that line the motorway junction.

**86.**  ***Punica granatum* L.**

Lythraceae—Archaeophyte—West Asia—Scapose phanerophyte

*Change in status for Calabria: from casual alien to naturalized alien (naturalized alien)*

*Specimina:* 7 March 2018, bank of Fiume Noce, Tortora (Cosenza), edge of the path in abandoned orchards and olive groves, 39.927421° N–15.76467° E, 8 m a.s.l., *leg. et det.* Fascetti S., Potenza G., Rosati L. (HLUC); 15 June 2020, bank of Fiume Abatemarco, S. Maria del Cedro (Cosenza), edge abandoned orchards, 39.757361° N–15.847366° E, 58 m a.s.l., leg. et det. Fascetti S., Potenza G., Rosati L. (HLUC); 5 July 2021, Via del Mare, Marcellina (Cosenza), edge of the path in abandoned orchards and olive groves, 39.757361° N–15.847366° E, 15 m a.s.l., *leg. et det.* Fascetti S., Potenza G., Rosati L. (HLUC).

Note. *Punica granatum* is reported for the region as a casual alien, but without a precise location. It is present in all Italian regions as a naturalized or casual alien, except in Toscana, where it is considered a dubious alien [43]. In north-west Calabria, the species is traditionally cultivated in mixed orchards with fig, olive and almond trees. By animals (zoochoria), it easily spreads to nearby moist soils at the edges of abandoned fields, forming dense hedges. A similar situation occurs in South-East Sicilia [148].

**87.**  ***Reynoutria ×bohemica* Chrtek & Chrtková** [≡*Fallopia bohemica* (Chrtek & Chrtková) J.P.Bailey; ≡ *Polygonum bohemicum* (Chrtek & Chrtková) Zika & Jacobson = *Reynoutria japonica* Houtt. × *Reynoutria sachalinensis* (F.Schmidt) Nakai]

Polygonaceae—Neophyte—Japan—Rhizomatose geophyte

*Change in status for Liguria: from casual alien to naturalized alien (naturalized alien)*

*Specimina:* 23 June 2005, road between Sciarborasca and Lerca (Via di Valverde), Cogoleto (Genova), impluvium, downstream side, ruderal margin, 44.407394° N–08.631026° E, 179 m a.s.l., S, *leg. et det.* F. Verloove 6006 (FI, LG); 29 August 2007, *ibidem*, *leg. et det.* G. Galasso (MSNM Nos. MSNM43249, MSNM43250, MSNM43251, MSNM43252, MSNM43253, MSNM43254); 27 June 2023, between Sciarborasca and Lerca, Via Colombo (strada SP78), Cogoleto (Genova), just downstream of the road, grassy escarpment, 44.406885° N–08.630824° E, 169 m a.s.l., SE, *leg.* G. Galasso, *det.* G. Galasso, E. Banfi (MSNM barcodes MSNM52537, MSNM52538, MSNM52539, MSNM52540).

Note. The same population observed in 2005 and 2007 [119] has been maintained by expanding along the slope.

**88.**  ***Robinia pseudoacacia* L.**

Fabaceae—Neophyte—East South U.S.A.—Scapose phanerophyte

*Change in status for Sardegna from naturalized alien to invasive alien (invasive alien)*

*Observatum:* 31 July 2023, Aradoni, Belvì (Nuoro), forestry habitats, 39.974433° N–09.187147° E, 602 m a.s.l., *leg. et det.* G. Bacchetta, G. Calvia, F. Mascia.

Note. *Robinia pseudoacacia* is a North American tree that has become a naturalized or invasive alien species in most European countries [80]. In Sardegna, in recent years, the species has become invasive alien in deciduous forests, particularly in the Sarcidano and Barbagia subsectors. The most affected habitats include riparian woodlands dominated by alder [*Alnus glutinosa* (L.) Gaertn.] and oak forests primarily composed of *Quercus dalechampii* Ten. and *Q. ichnusae* Mossa, Bacch. & Brullo (both species now included under *Quercus pubescens* Willd. subsp. *pubescens*). In other parts of Sardegna, *R. pseudoacacia* is mainly confined to roadsides and railroads.

**89.**  ***Ruellia simplex* C. Wright**

Acanthaceae—Neophyte—America—Nanophanerophyte

*First record for Calabria (casual alien)*

*Specimen:* 9 September 2022, via Argine Destro Calopinace, Reggio Calabria, ruderal site along the road, 38.101381° N–15.638202° E, 15 m a.s.l., *leg.* A. Capuano, *det.* A. Capuano, G. Caruso (REGGIO, Herb. Capuano).

*Observatum:* 30 October 2023, Condera, Reggio Calabria, abandoned area of the city cemetery, 38.106861° N–15.662997° E, 129 m a.s.l., *obs.* V.L.A. Laface.

Note. *Ruellia simplex* has been reported to Italy only for Sardegna, Puglia, Lazio [23,94,149] and recently for Sicilia [38]. A small population has been observed along the roadside, in a ruderal site where other alien species (such as *Cenchrus setaceus* (Forssk.) Morrone, *Amaranthus viridis* L. and *Salvia ×floriferior* Dolat. & Ziel.) occur. Seeds or plant fragments could have dispersed from individuals cultivated in neighboring areas. *Ruellia simplex* is considered an invasive alien species in the South-East U.S.A., Australia and Pacific archipelagos [150].

**90.**  ***Salvia ×floriferior* Dolat. & Ziel.** [≡*Salvia abrotanoides* (Kar.) Sytsma × *Salvia yangii* B.T.Drew]

Lamiaceae—Neophyte—Artificial hybrid (Culton)—Scapose hemicryptophyte

*First record for Calabria (casual alien)*

*Specimen:* 9 September 2022, via Argine Destro Calopinace, Reggio Calabria, ruderal site along the road, 38.101379° N–15.638146° E, 15 m a.s.l., *leg.* A. Capuano, *det.* A. Capuano, G. Caruso (REGGIO, Herb. Capuano).

Note. The genus *Perovskia* (Kar.) J.B.Walker, B.T.Drew & J.G.González, later reduced at the subgeneric rank and included into *Salvia* [151], counts about eight species distributed over arid regions of Asia [125], whose identification and nomenclature are complicated by several hybrids and cultivars selected by gardeners. Considering the morphological features of leaves, different from the supposed parental species, the collected material has been ascribed to the hybrid *S. abrotanoides × S. yangii* according to Galasso et al. [25] and Dolatowski et Zieliński [152]. The nomenclatural combination we report here is in accordance with IPNI [153]. Plants found in this first Calabrian site probably originated from material cultivated in the surroundings.

**91.**  ***Soehrensia spachiana* (Lem.) Schlumpb.**

Cactaceae—Neophyte—Argentina and Bolivia—Succulent chamaephyte

*First record for Calabria (casual alien)*

*Specimina:* 21 April 2022, Condofuri Marina, Condofuri (Reggio Calabria), synanthropic recovery vegetation of highly altered and disturbed environments, 37.928505° N–15.886612° E, 8 m a.s.l., *leg. et det.* G. Mei (Herbarium Mei); 25 January 2022, Via Sbarre, Reggio Calabria, synanthropic and ruderal vegetation for the recovery of highly altered and disturbed environments, 38.088493° N–15.643762° E, 21 m a.s.l., *leg.* G. Mei, G. Posillipo, *det.* G. Mei (Herbarium Mei).

Note. A partially rotting adult individual and a couple of young individuals of *S. spachiana* were found near a large pile of waste and disused objects inside a former grassland now overgrown with tall grass, outside the perimeter wall of a camping. The species was found in a disused area, completely incorporated into an urban area and colonized by synanthropic vegetation. In addition to the oldest individual most likely deriving from an old house plant disused and thrown away in the area, other individuals of different ages and sizes are observed below, given the arrangement probably deriving from root shoots of the older specimen.

**92.**  ***Solanum pseudocapsicum* L.**

Solanaceae—Neophyte—South America—Nanophanerophyte

*First record for Calabria (casual alien)*

*Specimen:* 5 May 2022, Catona, Reggio Calabria, synanthropic recovery vegetation of highly altered and disturbed environments, 38.199610° N–15.654890° E, 78 m a.s.l., *leg.* G. Mei, L. Manti, *det.* G. Mei (Herbarium Mei).

Note. A couple of individuals of *S. pseudocapsicum* of different ages and sizes, of which only one with flowers and fruits still present, were found inside a *fiumara*, on the edge of an illegal landfill bordered on three sides by reeds in *Arundo donax* L. and on one side by flooded section of the *fiumara*.

**93.**  ***Sorghum bicolor* (L.) Moench subsp. *bicolor***

Poaceae—Archaeophyte—Central Africa—Scapose therophyte

*First record for Calabria (casual alien)*

*Specimina:* 10 July 2023, Borgata Sant’Elia, Montebello Jonico (Reggio Calabria), unauthorized landfill with storage of waste materials, 37.926750° N–15.740278° E, 3 m a.s.l., *leg.* G. Mazzacuva, *det.* V.L.A. Laface, G. Mazzacuva (REGGIO); 1 November 2023, Gallico, Reggio Calabria, roadside, 38.171306° N–15.663694° E, 60 m a.s.l., *leg.* G. Mazzacuva, *det.* V.L.A. Laface, G. Mazzacuva (REGGIO).

Note. *Sorghum bicolor* is the fifth-most-produced cereal in the world and it is a source of nutrients and bioactive compounds for the human diet [154]. It is also used as an efficient biomass accumulator and has potential use as a cellulosic biofuel [155]. In Italy, it is now reported in all regions except Molise, Liguria and Valle d’Aosta [43].

**94.**  ***Sorghum halepense* (L.) Pers.**

Poaceae—Archaeophyte—North Africa, Arabian Peninsula and Central South Asia—Rhizomatose geophyte

*Change in status for Umbria: from casual alien to invasive alien (invasive alien)*

*Observatum:* 18 September 2022, Via Trasimeno I, Castiglione del Lago (Perugia), riparian vegetation, fields, ruderal sites, 43.166853° N–12.015372° E, 259 m a.s.l., *obs.* E. Fanfarillo.

Note. *Sorghum halepense* is widespread in fields, shores and ruderal sites in the area, so it should be considered an invasive alien for Umbria like in most Italian regions [43,156].

**95.**  ***Spinacia oleracea* L. subsp. *oleracea***

Amaranthaceae—Archaeophyte—Kazakhstan, Turkmenistan and Uzbekistan (Culton)—Scapose therophyte

*First record for Sicilia (casual alien)*

*Specimen:* 25 April 2017, Contrada Giuranella, Agrigento, roadside, 37.345555° N–13.543885° E, 240 m a.s.l., *leg.* G. Domina, *det.* G. Domina, E. Di Gristina, G. Barone (SAF100128).

Note. *Spinacia oleracea* subsp. *oleracea* is a culton domesticated from *Spinacia oleracea* subsp. *turkestanica* (Iljin) Del Guacchio & P. Caputo, widely used as a food plant. Some individuals have been found along a farm road, along the fields where the plant is cultivated. *Spinacia oleracea* subsp. *oleracea* is present in Italy as a casual alien in Lombardia, Veneto, Emilia-Romagna, Toscana, Marche, Umbria, Lazio, Abruzzo and Sardegna [43].

**96.**  ***Stenotaphrum secundatum* (Walter) Kuntze**

Poaceae—Neophyte—Central and South America, East U.S.A., Central West Africa—Rhizomatose geophyte

*Change in status for Sicilia: from casual to naturalized alien (naturalized alien)*

*Specimen:* 20 July 2023, Sant’Agata li Battiati (Catania), roadside, 37.56469° N–15.08221° E, 345 m a.s.l., *leg.* S. Cambria, G. Tavilla, *det.* S. Cambria (CAT).

Note. According to the Portal to the Flora of Italy [43], *S. secundatum* can be found as a casual alien species in Basilicata, Calabria, Campania, Liguria, Puglia, Sardegna and Sicilia. Moreover, it has only been observed as a naturalized alien in Toscana at present. Our recent research in Sicilia has revealed that this species is gradually spreading and becoming a naturalized alien in various areas of the island. Hence, it is crucial to update the status of this species to naturalized alien.

**97.**  ***Styphnolobium japonicum* (L.) Schott** [≡*Sophora japonica* L.]

Fabaceae—Neophyte—China—Scapose phanerophyte

*First record for Calabria (casual alien)*

*Specimen:* 14 August 2022, Pellaro, Reggio Calabria, in a crack of a small artificial water flow basin along the road, 38.014814° N–15.637740° E, 15 m a.s.l., *leg.* C.M. Musarella, *det*. V.L.A. Laface, C.M. Musarella, G. Spampinato (REGGIO).

*Observata:* 11 October 2022, Reggio Calabria, in the cracks in the sidewalks close to adult individuals, 38.119990° N–15.654580° E, 29 m a.s.l., *obs.* C.M. Musarella; 11 October 2022, Reggio Calabria, in the cracks in the sidewalks close to adult individuals, 38.117361° N–15.656277° E, 36 m a.s.l., *obs.* C.M. Musarella.

Note. *Styphnolobium japonicum* was already reported as a casual alien species in several Italian regions [43]. The collected specimen was found with two other juveniles probably coming from an adult tree located not far away, found together with other alien species. Other juveniles and seedlings were observed in some streets of downtown Reggio Calabria, close to planted adult individuals.

**98.**  ***Tecoma stans* (L.) Juss. ex Kunth**

Bignoniaceae—Neophyte—America—Caespitose phanerophyte

*First record for Sardegna (casual alien)*

*Specimen:* 20 August 2023, Rio Concias, S. Sperate (Sud Sardegna), synanthropic habitats, 39.357839° N–09.004965° E, 37 m a.s.l., *leg. et det.* L. Podda, F. Mascia, G. Bacchetta (CAG).

Note. A few individuals of *T. stans* were found along the artificial riverbanks of Rio Concias (S. Sperate, Sud Sardegna), and probably escaped from confining gardens. In Italy, this species is only documented in Calabria, where it was first reported in Europe [157].

**99.**  ***Tradescantia pallida* (Rose) D.R.Hunt**

Commelinaceae—Neophyte—Mexico—Rhizomatose geophyte

*First record for Basilicata (casual alien)*

*Specimen:* 25 June 2023, Contrada Molino Rosa, Lauria (Potenza), road slope, 40.050966° N–15.820988° E, 412 m a.s.l., *leg. et det.* Fascetti S., Potenza G., Rosati L. (HLUC).

Note. *Tradescantia pallida* was reported recently as a casual alien of synanthropic and urbanized environments in the neighboring regions of central-southern Italy [34,52,66]. The species is widespread as an ornamental plant and it could potentially become a naturalized alien due to its lively vegetative reproduction.

**100.**  ***Triticum aestivum* L. subsp. *spelta* (L.) Thell.**

Poaceae—Archaeophyte—Transcaucasus—Scapose therophyte

*First record for Sardegna (casual alien)*

*Specimen:* 19 July 2021, Strovina, Sanluri (Sud Sardegna), agricultural and rural habitats, 39.524342° N–08.842611° E, 56 m a.s.l., *leg. et det.* F. Mascia, L. Podda, G. Bacchetta (CAG).

Note. *Triticum aestivum* subsp. *spelta* is occasionally cultivated in Sardegna. Several individuals were found as weeds in the cultivated fields of durum and bread wheat. This taxon is only reported for Campania as a casual alien [51].

**101.**  ***Triticum turgidum* L. subsp. *dicoccon* (Schrank ex Schübl.) Thell.**

Poaceae—Archaeophyte—Turkey—Scapose therophyte

*First record for Sardegna (casual alien)*

*Specimen:* 25 June 2021, Sa Piedadi, San Gavino Monreale (Sud Sardegna), agricultural and rural habitats, 39.554330° N–08.749300° E, 50 m a.s.l., *leg. et det.* F. Mascia, G. Calvia, G. Bacchetta (CAG).

Note. *Triticum turgidum* subsp. *dicoccon* is increasingly cultivated in Sardegna and is commonly found as a weed in durum wheat cultivation. This taxon is documented as a casual alien in three other regions of Italy: Campania [93], Lombardia [50] and Toscana [158].

**102.**  ***Ulmus pumila* L.**

Ulmaceae—Neophyte—Central Asia—Caespitose phanerophyte

*First record for Umbria (casual alien)*

*Specimen*: 23 December 2022, Orvieto Scalo, Orvieto (Terni), ruderal habitats, 42.715318° N–12.146131° E, 111 m a.s.l., *leg. et det.* E. Fanfarillo (SIENA).

Note. A few individuals of *U. pumila* were found at the bottom of a wall. This species is currently spreading in central Italy, where it is widely naturalized [35,58]. This species is naturalized as well in many regions of northern Italy and in Puglia, while it is considered an invasive alien in Piemonte and Valle d’Aosta [43] and a casual alien in Calabria [7].

**103.**  ***Vicia lens* (L.) Coss. & Germ. subsp. *lens*** [≡*Ervum lens* L.]

Fabaceae—Archaeophyte—South West Asia (Culton)—Scapose therophyte

*First record for Sicilia (casual alien)*

*Specimen:* 12 April 2023, Punta Gorgo Salato, Ustica (Palermo), uncultivated land, 38.720035° N–13.180388° E, 10 m a.s.l., *leg.* G. Domina, *det.* G. Domina, E. Di Gristina, G. Barone (SAF100130).

Note. Culton domesticated from *Vicia lens* subsp. *orientalis* (Boiss.) Galasso, Banfi, Bartolucci & J.-M.Tison, *V. lens* subsp. *lens* is widely used as a food plant. Some individuals have been found in uncultivated land, where the plant was presumably grown in previous years. This taxon is present throughout Italy as a casual alien and is not reported only in Valle d’Aosta, Veneto, Molise, Basilicata and Calabria [43].

**104.**  ***Vitis riparia* Michx.**

Vitaceae—Neophyte—North America—Climbing phanerophyte

*First record for Umbria (casual alien)*

*Specimen:* 3 June 2023, Casalina, Deruta (Perugia), riparian vegetation, 42.953506° N–12.395102° E, 162 m a.s.l., *leg. et det.* T. Fiaschi (SIENA).

Note. *Vitis riparia* grows within riparian vegetation along the Tiber river. It is present in 10 Italian regions and it is considered invasive alien in Piemonte, Lombardia, Toscana, Marche and Abruzzo [43].

**105.**  ***Yucca gigantea* Lem.**

Asparagaceae—Neophyte—Central America and Mexico—Caespitose phanerophyte

*First record for Sardegna (casual alien)*

*Specimen:* 13 April 2022, Stani Saliu, Sestu (Cagliari), agricultural and rural habitats, 39.330941° N–09.091606° E, 52 m a.s.l., *leg. et det.* L. Podda, A. Lallai, G. Calvia (CAG).

Note. Some individuals of *Y. gigantea* close to Stani Saliu pond (Sestu, Cagliari) probably developed from waste material. Other individuals, always in small groups, were observed near S. Gilla and Molentargius wetlands (Cagliari).

**106.**  ***Zinnia elegans* Jacq.**

Asteraceae—Neophyte—Central America and Mexico—Scapose therophyte

*First record for Calabria (casual alien)*

*Specimina:* 21 November 2021, Strada Provinciale 6, Fiumara (Reggio Calabria), roadside, 38.209645° N–15.690341° E, 155 m a.s.l., *leg. et det.* V.L.A. Laface (REGGIO); 30 October 2021, Strada Provinciale Archi-Ortì, Ortì Inferiore, Reggio Calabria, roadside, 38.147913° N–15.708878° E, 606 m a.s.l., *leg. et det.* V.L.A. Laface (REGGIO); 19 September 2021, Amendolea, Condofuri (Reggio Calabria), roadside, 37.988167° N–15.893037° E, 163 m a.s.l., *leg. et det.* V.L.A. Laface (REGGIO); 26 September 2021, Villamesa di Calanna, Calanna (Reggio Calabria), roadside, 38.198231° N–15.714790° E, 436 m a.s.l., *leg. et det.* V.L.A. Laface (REGGIO).

Note. *Zinnia elegans* is native to Central America [61]. It is commonly used as an ornamental plant; the seedlings observed along the roadside probably came from nearby gardens and villas. *Zinnia elegans* is present in several Italian regions as a casual alien [43].

### 2.2. Italian Alien Flora Update

The current amount of the Italian alien flora [43] of 1659 specific and subspecific taxa (excluding taxa reported by mistake, doubtful species, data deficient, historical records, and extinct) is updated to 1666 thanks to the new records presented in this study. Of the seven new records for Italian alien flora, two are new for Europe (*Bauhinia variegata* L., *Pinus elliottii* Engelm.), one for continental Europe (*Persicaria senegalensis* (Meisn.) Soják), three for Italy (*Bauhinia purpurea* L., *Citrullus amarus* Schrad., *Cytisus proliferus* L.f. subsp. *proliferus*) and one for the Italian Peninsula (*Portulacaria afra* Jacq.).

The current amount of the Italian flora (native + alien, excluding the taxa reported by mistake, doubtful species, data deficient, historical records and extinct) including the new records reported in this study is 9303 taxa.

**Figure 1:**
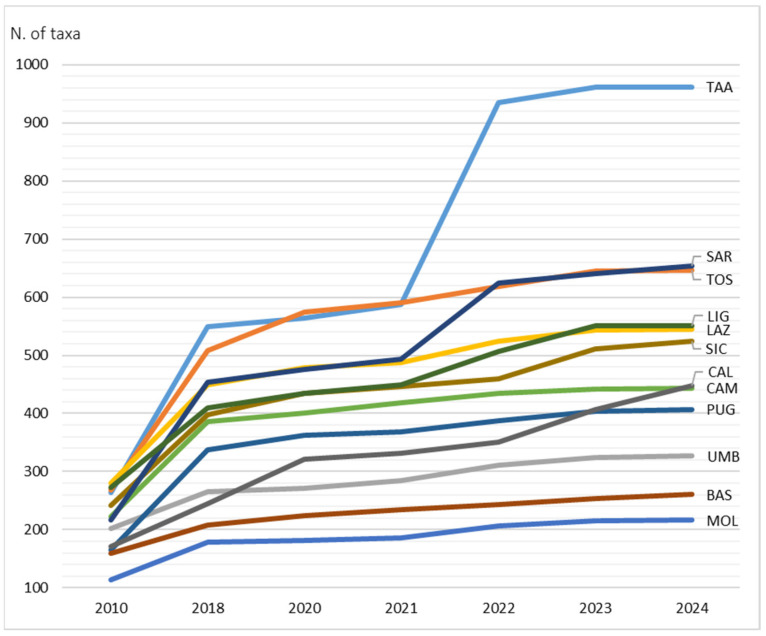
Increase in the number of alien taxa, for the 12 Italian regions surveyed with this study, from 2010 to present. (BAS: Basilicata; CAL: Calabria; CAM: Campania; LAZ: Lazio; LIG: Liguria; MOL: Molise; PUG: Puglia; SAR: Sardegna; SIC: Sicilia; TOS: Toscana; TAA: Trentino-Alto Adige; UMB: Umbria).

Comparing the contributions of various Italian authors [27,43,58,159,160,161] over the past 14 years with the data provided by this study, there is a steady increase in allochthonous taxa (Table 3, Figure 1).

A steady increase in alien taxa is observed in all Italian regions, some of which, such as Trentino Alto Adige and Sardegna, are over 200%. The average percentage increase for Italian regions is more than 100% (Table 4).

The percentage of alien flora in the various regions (Degree of floristic pollution) varies between 7.6% in Valle d’Aosta, to 23.5% in Lombardia and Trentino Alto Adige, with an average value for the Italian territory of 19.9% (Table 4).

**Figure 2:**
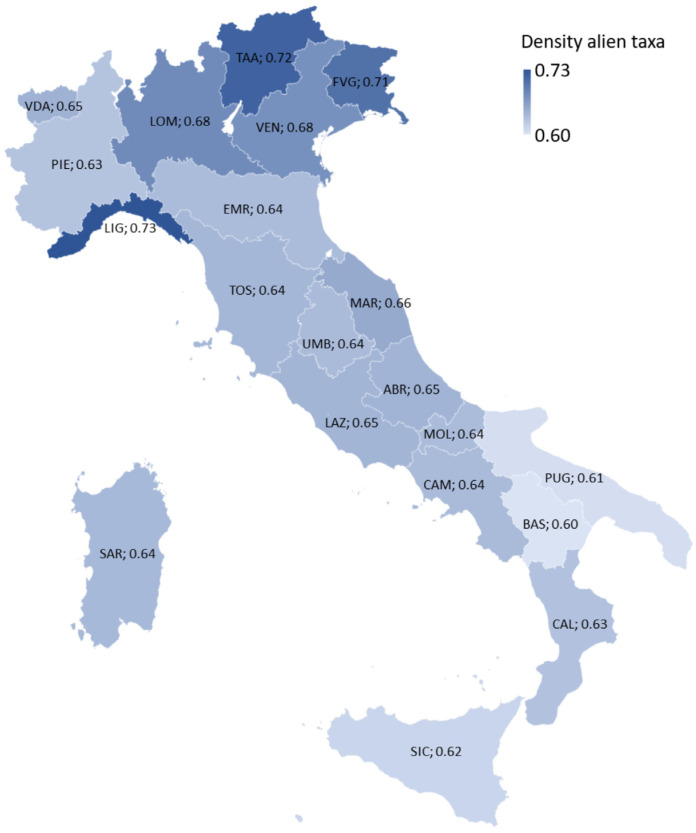
Density of alien taxa (Ln N. taxa/Ln km^2^) for the italian regions (ABR: Abruzzo; BAS: Basilicata; CAL: Calabria; CAM: Campania; EMR: Emilia-Romagna; FVG: Friuli Venezia Giulia; LAZ: Lazio; LIG: Liguria; LOM: Lombardia; MAR: Marche; MOL: Molise; PIE: Piemonte; PUG: Puglia; SAR: Sardegna; SIC: Sicilia; TOS: Toscana; TAA: Trentino-Alto Adige; UMB: Umbria; VDA: Valle d’Aosta; VEN: Veneto).

This value is not really significant for the area surveyed unless the surface is taken into account, thus assessing the density of alien taxa, which is highest in regions of north Italy such as Liguria (0.73), Trentino Alto Adige (0.72), Friuli Venezia Giulia (0.71), Veneto and Lombardia (0.68), while it is minimal for southern regions such as Basilicata (0.60) and Puglia (0.61) (Figure 2).

**Figure 3:**
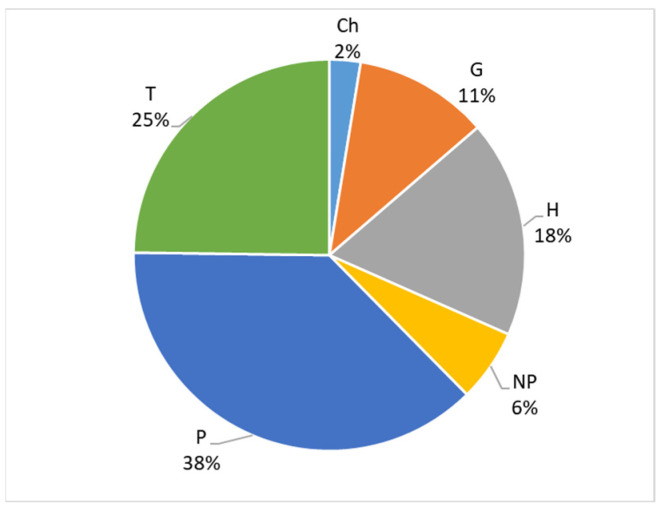
Life form spectrum of updated alien species (Ch: Chamaephyte; G: Geophyte; H: Hemicryptophyte; NP: Nanophanerophyte; P: Phanerophyte; T: Therophyte).

The analysis of the life forms of the 106 alien taxa added to the Italian flora in this contribution shows that phanerophytes correspond to 38%, followed by therophytes with 25%, and hemicryptophytes with 18%, with the other life forms having smaller percentages (Figure 3). The predominance of phanerophytes is most probably due to the fact that these are mostly species introduced for ornamental purposes as *Bauhinia variegata* L., *Pinus elliottii* Engelm., *Bauhinia purpurea* L., *Styphnolobium japonicum* (L.) Schott, *Chamaecyparis lawsoniana* (A.Murray) Parl., *Jacaranda mimosifolia* D.Dono, for agronomic interest as *Punica granatum* L., *Annona cherimola* Mill., *Actinidia deliciosa* (A.Chev.) C.F.Liang & A.R.Ferguson, *Diospyrus lotus* L., *Diospyrus virginiana* L. or forestry *Cedrus atlantica* (Endl.) G.Manetti ex Carrière. Therophytes were partly accidentally introduced as *Euphorbia serpens* Kunth subsp. *serpens*, *Cenchrus longispinus* (Hack.) Fernald, *Moeroris tenella* (Roxb.) R.W.Bouman, some are of agronomic interest that have escaped cultivation such as *Cucumis melo* L. subsp. *melo*, *Cicer arietinum* L. subsp. *arietinum*, *Sorghum bicolor* (L.) Moench subsp. *bicolor*, *Spinacia oleracea* L. subsp. *oleracea*, *Triticum aestivum* L. subsp. *spelta* (L.) Thell., *Triticum turgidum* L. subsp. *dicoccon* (Schrank ex Schübl.) Thell., *Vicia lens* (L.) Coss. & Germ. subsp. lens, *Ocimum basilicum* L., others were introduced for ornamental purposes such as *Gibasis pellucida* (M.Martens & Galeotti) D.R.Hunt, *Impatiens balsamina* L., *Lobelia erinus* L., *Perilla frutescens* (L.) Britton, *Zinnia elegans* Jacq.

## 3. Materials and Methods

Newly introduced alien plants in the Italian and European territories and changes in the invasiveness status of species already present in the territory were considered here, in accordance with Pyšek et al. [55]. The specimens collected and examined are deposited in the following public herbaria registered in the Index Herbariorum (according to Thiers [162]: herbarium of the Mediterranean University of Reggio Calabria (Italy) (REGGIO); central herbarium of Firenze (FI); Herbarium of Johannes Gutenberg-Universität in Mainz (Germany) (MJG); Herbarium of Catania University of Catania (Italy) (CAT); Herbarium of University of Campania Luigi Vanvitelli, Herb. Austroitalicum (Italy) (IT); Napoli (Italy) (PORUN); Milan Natural History Museum (Italy) (MSNM); University of Liège (Belgium); Herbarium Lucanum in University of Basilicata, Potenza (Italy) (HLUC); Herbarium of University of Siena (Italy) (SIENA); Herbarium of Department of Agricultural and Forest Sciences, Palermo (Italy) (SAF); Herbarium of University of Napoli, Federico II, Napoli (Italy) (NAP); Herbarium of University of Messina (Italy) (MS); and Herbarium of University of Cagliari (Italy) (CAG). Moreover, some private collections are considered: Herbarium R.P. Wagensommer; Herb. Mei; Herb. Capuano; Herb. Calvia; Herb. N. Biscotti; Herb. D. Bonsanto.

The floristic list of the taxa is reported in alphabetical order and follows the updated nomenclature and regional distribution of the checklist of the Italian vascular flora available on the Portal to the Flora of Italy [43], referable to the checklist of the flora alien to Italy [27] and subsequent updates [28,29,30,31,32,33,34,35,36,37,38,39].

The collected taxa were identified in accordance with the Flora d’Italia [163,164,165,166,167], Flora Europaea [168,169,170,171,172], Flora of North America [173], and Flora of China [174]. Other Italian regional floras were consulted [158,175,176,177,178,179,180,181].

Life forms, according to Raunkiaer classification [182], followed Pignatti [163,164,165,166,167].

The following information is provided for each taxon: updated scientific name, basionym (if present) and most relevant synonyms obtained from consultation of the sites Portal to the Flora of Italy [43] and IPNI [153]; family and biological form according to Flora d’Italia [167] [also available at IPFI: Index Plantarum of Acta Plantarum [183]; the range of origin according to POWO [61] and EPPO [184], period of introduction in Italy (i.e., archaeophyte or neophyte) according to “Portal to the Flora of Italy” [43], the invasiveness status (according to [55]) and whether the species is reported for the first time at regional, Italian or European level or whether a change in status has occurred.

For each taxon reported, the place of discovery and if the sample is a *specimen* or more *specimina* (with deposited herbarium *specimen* or *specimina*), *observatum* or more *observata* (species observed growing in the wild) are also indicated. The following specific information on the collection or observation site(s) is also included: toponym (according to the “Geoportale Nazionale” [185], locality, municipality, province or metropolitan city (either of the latter two are indicated in parentheses only when they do not correspond to the name of the provincial capital municipality), geographical coordinates N and E (datum WGS84, UTM) and altitude. The metropolitan cities considered in this work are as follows: Bari, Cagliari, Catania, Firenze, Genova, Messina, Napoli, Palermo, and Reggio Calabria; all the others are provinces. Moreover, the growing environment(s), date of collection/observation, collector(s) (*legit: leg.*), observer(s) (*observavit: obs.*), author(s) of the identification (*determinavit: det.*). If the observer and the author of the identification are the same person, only “*obs.*” is reported. If they are different persons, “*obs.*” and “*det.*” are reported.

In order to carry out a comparison across Italian regions, the percentage increase in alien taxa from 2010 to the present was evaluated, taking all Italian regions into consideration. To this end, the works carried out by several authors [27,43,58,159,160,161] were considered and compared with the new data presented in this study. In addition, two indices were calculated: the Degree of floristic pollution (%), expressing the percentage of alien taxa in the total flora of the area (native + alien taxa, excluding those reported by mistake, doubtful species, data deficient, historical records, and extinct) and the Density alien taxa, expressing the ratio of the natural logarithm of the number of alien taxa to the natural logarithm of the surface of the examined area (Ln N. alien taxa/Ln km^2^). For the surfaces of Italian administrative regions, reference is made to updated ISTAT data [186].

## 4. Conclusions

As many as 106 new taxa between first reports, status changes and extinctions for 11 of Italy’s 20 administrative regions represent a significant increase in the number of alien species known to date for the Italian territory. Some of them also represent novelties for Italian and European territory. This study confirms the constant increase in alien species in the flora of the Italian territory: a common trend on a global scale. These results should further warn against biological invasions that still do not find a sufficient barrier to stop their advance. Floristic studies and field research such as this one and the others considered in this paper for the Italian territory demonstrate once again how knowledge of flora is a basic tool for biodiversity preservation.

## Figures and Tables

**Table 1:** New floristic records of alien taxa for the 12 Italian regions surveyed.

Italian Regions	Change in Status	Extinct	First Record	Total
Basilicata			8	8
Calabria	7		41	48
Campania	3		2	5
Lazio			1	1
Liguria	3			3
Molise			1	1
Puglia	3	1	5	9
Sardegna	4		13	17
Sicilia	2		13	15
Trentino Alto Adige			1	1
Toscana	3		1	4
Umbria	2		3	5
**Total records**	**27**	**1**	**89**	**117**

**Table 2:** Invasive status of the alien taxa recorded for the 12 Italian regions surveyed.

Italian Regions	CAS	NAT	INV	EX
Basilicata	8			
Calabria	39	6	3	
Campania	2	2	1	
Lazio	1			
Liguria		3		
Molise	1			
Puglia	3	5		1
Sardegna	12	1	4	
Sicilia	12	3		
Trentino Alto Adige	1			
Toscana		2	2	
Umbria	2	1	2	
**Total records**	**81**	**23**	**12**	**1**

**Table 3:** Consistency of alien flora from 2010 to present for the 12 Italian regions surveyed with this study. In detail, the works consulted and the reference year of the data considered: Celesti Grapow et al. [159]: 2010; Galasso et al. [27]: 2018; Bartolucci et al. [160]: 2020; Bartolucci et al. [161]: 2021; Stinca et al. [58]; Portal to the Flora of Italy [43]: 2023; data updated with the present study: 2024.

Italian Regions	2010	2018	2020	2021	2022	2023	2024
Trentino Alto Adige	264	549	564	588	935	961	962
Toscana	268	508	574	590	618	645	646
Umbria	202	265	271	284	311	324	327
Lazio	280	450	479	487	525	544	545
Molise	114	178	182	186	206	215	216
Campania	222	386	400	419	435	442	444
Puglia	165	337	362	368	387	404	407
Basilicata	160	208	224	235	243	253	261
Calabria	171	244	321	332	350	407	448
Sicilia	242	398	434	447	460	511	524
Sardegna	216	453	476	494	625	641	654
Liguria	273	409	435	449	506	551	551

**Table 4:** Surface, percentage increase in alien taxa, degree of floristic pollution, and density of alien taxa in the 20 regions of Italy.

Italian Regions	Surface (km^2^)	Increase Alien Taxa from 2010 to 2024 (%)	Degree of Floristic Pollution (%)	Density Alien Taxa (Ln N. taxa/Ln km^2^)
Abruzzo	10,831.50	89.3	11.3	0.65
Basilicata	10,073.11	63.1	9.1	0.60
Calabria	15,221.61	162.0	14.2	0.63
Campania	13,670.60	100.0	13.5	0.64
Emilia-Romagna	22,501.43	101.0	17.8	0.64
Friuli Venezia Giulia	7932.48	81.8	15.8	0.71
Lazio	17,231.72	94.6	15.2	0.65
Liguria	5416.15	101.8	15.4	0.73
Lombardia	23,863.10	83.6	23.5	0.68
Marche	9344.29	66.8	13.6	0.66
Molise	4460.44	89.5	8.6	0.64
Piemonte	25,386.70	88.9	16.0	0.63
Puglia	19,540.52	146.7	13.7	0.61
Sardegna	24,099.45	202.8	22.1	0.64
Sicilia	25,832.55	116.5	16.1	0.62
Toscana	22,987.44	264.4	15.7	0.64
Trentino-Alto Adige	13,604.72	141.0	23.5	0.72
Umbria	8464.22	61.9	12.1	0.64
Valle d’Aosta	3260.85	128.9	7.6	0.65
Veneto	18,345.37	115.3	19.7	0.68
ITALY	302,073	73.4	19.9	0.59

## Data Availability

Data are contained within the article and Supplementary Materials.

## Supplementary Materials

The following supporting information can be downloaded at: https://www.mdpi.com/article/10.3390/plants13050620/s1, Table S1: Record list of new alien taxa for Italian flora.
